# The 100 most-cited articles in COVID-19: a bibliometric analysis

**DOI:** 10.1093/eurpub/ckae098

**Published:** 2024-07-06

**Authors:** Yong Y Liew, Qiming Dong, Nivan Lakshman, Ankur Khajuria

**Affiliations:** Nuffield Department of Surgical Sciences, Kellogg College, University of Oxford, Oxford, United Kingdom; Department of Internal Medicine, Greater Baltimore Medical Center, Towson, MD, United States; Medical College of Georgia, Augusta, GA, United States; Nuffield Department of Surgical Sciences, Kellogg College, University of Oxford, Oxford, United Kingdom; Department of Surgery and Cancer, Imperial College London, London, United Kingdom

## Abstract

Corona virus disease 2019 (COVID-19) pandemic, sparked by the emergence of a novel coronavirus in early 2020, has prompted a surge in published articles. This study aims to systematically analyse the characteristics and trends of impactful research in the field. The 100 most-cited publications associated with COVID-19 were identified by two independent reviewers using the ‘Web of Science’ database across all available journals up to the year 2023. Data collected include country, citation count, subject, level of evidence (using Oxford Centre for Evidence-Based Medicine System 2011), impact factor, funding, and study design. We identified 394 038 publications, and the 100 most-cited publications were ranked. These were cited by a total of 283 034 articles (median citation = 767), median impact factor of 66.9 and 72 articles with fundings. China (*n* = 44), USA (*n* = 19), and UK (*n* = 13) were the three highest contributors (*n* = 220 505). Most articles were level 5 evidence (*n* = 48), followed by level 3 (*n* = 28), 4 (*n* = 14), 2 (*n* = 7), and 1 (*n* = 3). The main subjects were mechanism of action and structures of SARS-CoV-2 virus (*n* = 18) and impact of COVID-19 on public health (*n* = 18). Publications in 2022 and 2023 predominantly focused on the impact of COVID-19. Majority of the highly cited studies were of low-to-moderate quality, with only 10 consisting of randomized controlled trials or systematic reviews with or without meta-analysis. These findings reflect a growing interest in understanding the impact of COVID-19 pandemic on public and mental health. This analysis found the potential for future double-blinded randomized controlled trials to validate existing findings.

## Introduction

In early 2020, the novel coronavirus SARS-CoV-2, known as COVID-19, emerged in Wuhan, China, causing a global public health emergency [1]. Efforts to contain the virus through awareness, lockdowns, and testing have been widespread. Despite these measures, COVID-19 has had significant negative socioeconomic and health impacts [2]. The global scientific community has responded to this public health emergency by participating in extensive research and publications to elucidate the viral mechanism, prevention techniques, update management strategies, epidemiological study, diagnostic tools, and risk stratification [3]. According to Organization for Economic Co-operation and Development (OECD), research funding for COVID-19 has reached 7 billion dollars to address the abovementioned issues [4].

The COVID-19 outbreak has led to an exponential increase in published articles. Škorić *et al*. [3] found 5761 COVID-19-related articles on PubMed as of April 2020. This surge in publications reflects the urgent need for timely data in managing the pandemic. However, the rapid increase may compromise the quality of information due to expedited reviews. Therefore, we aim to conduct a comprehensive bibliometric analysis of the top 100 most-cited articles on COVID-19 to evaluate their characteristics, quality, and trends.

## Methods

We have undertaken a comprehensive literature search to identify the 100 most-cited articles on COVID-19. We searched the online database—Web of Science (Clarivate Analytics, Philadelphia, Pa)—using the terms ‘COVID-19’ OR ‘coronavirus-19’ OR ‘SARS-CoV 2’ OR ‘coronavirus disease 2019’ as a ‘topic’ on 18 February 2023, 1930 (GMT zone). The period of the search includes all the available years up until 2023.

Of the publications gathered from Web of Science, we ranked these in descending order of ‘times cited’. Articles with an equal number of citations were separated by the average number of citations per year, with the more recent articles ranking higher.

**Figure 1.**
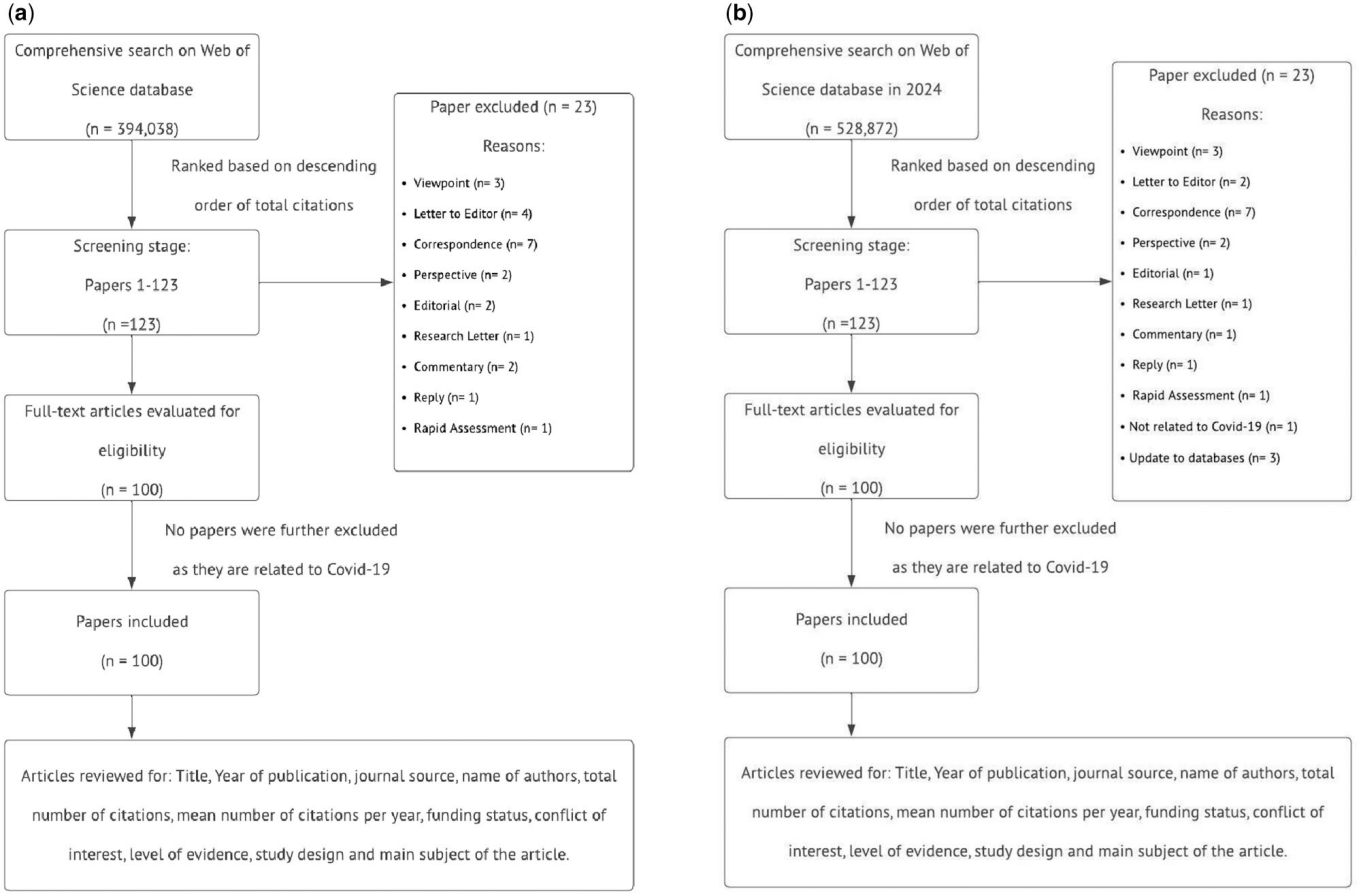
(a) Flowchart showing the methodology for article screening and data extraction for the 2023 search. (b) Flowchart showing the methodology for article screening and data extraction for the 2024 search 98 × 69 mm (300 × 300 DPI).

Two reviewers (Y.L.L. and N.L.) independently reviewed these articles to obtain the 100 most-cited papers relevant to ‘Covid-19’. Any discrepancies were resolved by the consensus discussion with another author (Q.M.D.). Further doubts were resolved by examining the article’s full text. A total of 123 papers were examined to provide 100 articles included in this study. Exclusion criteria include articles that were not related to COVID-19 and if the article was not in full-text form. Figure 1a shows a flowchart summarizing our methodology.

Following the screening stage, the data were independently extracted from the full texts by three authors (Y.L.L., N.L., and Q.M.D.). The data extracted were categorized into article title, year of publication, names of authors, source journal, the total number of citations, average number of citations per year, level of evidence, study design, funding status, conflict of interest, and the main subject of the article. We apply the Oxford Centre for Evidence-Based Medicine System 2011 to classify our level of evidence ranging from evidence level 1 (systematic review and meta-analysis), level 2 (randomized controlled trial), level 3 (non-randomized controlled cohort, retrospective and prospective cohort study), level 4 (case-series, case-control), and level 5 (laboratory studies, questionnaires, non-clinical studies, case report) [5]. These data were documented onto a standardized computer spreadsheet (Microsoft Excel, Version 16.71).

Another comprehensive literature search was carried out in April 2024 to assess for stability of the 100 most-cited articles one year following the initial search. The methodology is the same as the previous search with changes to the timeline of interest to include articles from inception to 2024. Figure 1b shows the flowchart summarizing our methodology.

## Results

### Initial search in 2023

Collectively, we gathered a total of 394 038 publications from the Web of Science in 2023. Of these, the top 100 most-cited papers associated with the theme of ‘Covid-19’ were ranked. (Please refer to Supplementary Appendix S1, which displays complete citations provided for all of the 100 most-cited articles associated with COVID-19.)

The paper with the highest citation number was cited by 16 248 articles, while the lowest citation number was 1508 as shown in Table 1. Collectively, the 100 most-cited articles on COVID-19 were cited by a total of 283 034 articles. The average number of citations per article per year ranged from 5416 to 469. The highest cited article belonged to a retrospective cohort study conducted by Guan W-Jie *et al*. [6] from China entitled ‘Clinical Characteristics of Coronavirus Disease 2019 in China’. The paper assessed the clinical presentation, incubation, and radiological diagnosis of COVID-19. The study had shown that the most common clinical presentation is fever followed by cough with a mean incubation period of four days. The most common radiological finding on computed tomography of the chest is ground glass changes. On the other hand, the lowest cited article in our study belonged to a narrative study conducted by Singhal [7] from India entitled ‘A Review of Coronavirus Disease-2019 (COVID-19)’. The paper discussed the course of COVID-19 outbreak and the record of COVID-19 associated death as of 2020. Tanu also discussed the measures taken by different countries to reduce the spread of COVID-19 during the peak of the pandemic.

**Table 1.** List of 100 most-cited articles on COVID-19 for the 2023 search

Rank	Authors	Journals	Impact factor	Total number of citations	Mean citations per year	Level of evidence	Country	Year of publication
1	Guan W. et al.	NEW ENGLAND JOURNAL OF MEDICINE	176.079	16 248	5416	3	China	2020
2	Zhou F. et al.	LANCET	202.731	14 592	4864	3	China	2020
3	Hoffmann M. et al.	CELL	66.85	10 234	3411.33	5	Germany	2020
4	Polack F.P. et al.	NEW ENGLAND JOURNAL OF MEDICINE	176.079	6733	2244.33	2	USA	2020
5	Long Q.X. et al.	NATURE MEDICINE	87.241	5853	1951	4	China	2020
6	The RECOVERY Collaborative Group	NEW ENGLAND JOURNAL OF MEDICINE	176.079	5720	2860	2	UK	2021
7	Bai Y. et al.	JAMA-JOURNAL OF THE AMERICAN MEDICAL ASSOCIATION	157.335	5413	1763.67	5	China	2020
8	Mao L. et al.	JAMA NEUROLOGY	11.5	5291	1763.67	4	China	2020
9	Walls A.C. et al.	CELL	66.85	4923	1641	5	USA	2020
10	Baden L.R. et al.	NEW ENGLAND JOURNAL OF MEDICINE	176.079	4488	2244	2	UK	2021
11	Wu C. et al.	JAMA INTERNAL MEDICINE	44.409	4459	1486.33	3	China	2020
12	Coronaviridae Study Group of the International Committee on Taxonomy of Viruses	NATURE MICROBIOLOGY	30.964	4404	1468	5	International	2020
13	Yang X. et al.	LANCET RESPIRATORY MEDICINE	102.642	4258	1419.33	3	China	2020
14	Beigel J.H. et al.	NEW ENGLAND JOURNAL OF MEDICINE	176.079	3718	1239.33	2	UK	2020
15	Richardson S. et al.	JAMA-JOURNAL OF THE AMERICAN MEDICAL ASSOCIATION	157.335	3632	1210.67	4	USA	2020
16	Lai J. et al.	JAMA NETWORK OPEN	13.37	3544	1181.33	4	China	2020
17	Klok F.A. et al.	THROMBOSIS RESEARCH	10.409	3393	1131	3	Netherlands	2020
18	Wang C. et al.	INTERNATIONAL JOURNAL OF ENVIRONMENTAL RESEARCH AND PUBLIC HEALTH	4.614	3319	1106.33	4	China	2020
19	Tao Ai et al.	RADIOLOGY	29.146	3260	1086.67	3	China	2020
20	Rani S. and Kumar R.	SPATIAL INFORMATION RESEARCH	2	3204	3204	5	India	2022
21	Ruan Q. et al.	INTENSIVE CARE MEDICINE	3.142	3181	1060.33	3	China	2020
22	Cao B., et al.	NEW ENGLAND JOURNAL OF MEDICINE	176.079	3148	1049.33	2	China	2020
23	Xu Z. et al.	LANCET RESPIRATORY MEDICINE	102.642	3131	1043.67	5	*China*	2020
24	Lauer S.A. et al.	ANNALS OF INTERNAL MEDICINE	51.598	3049	1016.33	3	USA	2020
25	Lan J. et al.	NATURE	69.504	3034	1011.33	5	China	2020
26	Gautret P. et al.	INTERNATIONAL JOURNAL OF ANTIMICROBIAL AGENTS	15.441	3016	1005.33	3	France	2020
27	Ackermann M. et al.	NEW ENGLAND JOURNAL OF MEDICINE	176.079	3008	1002.67	5	Germany	2020
28	Williamson E.J. et al.,	NATURE	69.504	2993	996.67	3	UK	2020
29	Qin C. et al.	CLINICAL INFECTIOUS DISEASES	20.999	2925	975	3	China	2020
30	Yan R. et al.	SCIENCE	47.73	2809	936.33	5	China	2020
31	Holmes E.A. et al.	LANCET PSYCHIATRY	77.056	2782	927.33	5	UK	2020
32	Liang W. et al.	LANCET ONCOLOGY	54.433	2678	892.67	3	China	2020
33	Nicola M. et al.	INTERNATIONAL JOURNAL OF SURGERY	13.4	2655	885	5	UK	2020
34	Chen G. et al.	JOURNAL OF CLINICAL INVESTIGATION	19.477	2637	879	3	China	2020
35	Lai C.C. et al.	INTERNATIONAL JOURNAL OF ANTIMICROBIAL AGENTS	15.441	2599	866.33	5	Taiwan	2020
36	Sohrabi C. et al.	INTERNATIONAL JOURNAL OF SURGERY	13.4	2576	858.67	5	UK	2020
37	Guan W. et al.	EUROPEAN RESPIRATORY JOURNAL	16.67	2534	844.67	3	China	2020
38	Dong Y. et al.	PEDIATRICS	9.703	2506	835.33	3	China	2020
39	Rothan H.A., Byrareddy S.N.	JOURNAL OF AUTOIMMUNITY	14.511	2496	832	5	USA	2020
40	WHO	PEDIATRIA I MEDYCYNA RODZINNA-PAEDIATRICS AND FAMILY MEDICINE	0.131	2417	805.67	5	International	2020
41	Shi S. et al.	JAMA CARDIOLOGY	30.17	2383	794.33	3	China	2020
42	Wiersinga W.J. et al.	JAMA-JOURNAL OF THE AMERICAN MEDICAL ASSOCIATION	157.335	2360	786.67	5	Netherlands	2020
43	Chen T. et al.	BMJ-BRITISH MEDICAL JOURNAL	93.333	2342	780.67	4	China	2020
44	Tay M.Z. et al.	NATURE REVIEWS IMMUNOLOGY	108.555	2335	778.33	5	Singapore	2020
45	Guo T. et al.	JAMA CARDIOLOGY	30.17	2311	770.33	4	China	2020
46	Cao W. et al.	PSYCHIATRY RESEARCH	11.225	2292	764	5	China	2020
47	Voiysey M. et al.	LANCET	202.731	2260	1130	2	International	2021
48	Guo Y.R. et al.	MILITARY MEDICAL RESEARCH	34.915	2221	740.33	5	China	2020
49	Blanco-Melo D. et al.	CELL	66.85	2191	741.67	5	USA	2020
50	Bavel J.J. et al.	NATURE HUMAN BEHAVIOUR	24.252	2171	723.67	5	USA	2020
51	Dhama K. et al.	CLINICAL MICROBIOLOGY REVIEWS	50.129	2146	715.33	5	India	2020
52	Korber B. et al.	CELL	66.85	2126	708.67	5	USA	2020
53	Chen H. et al.	LANCET	202.731	2054	684.67	4	China	2020
54	Xiong J. et al.	JOURNAL OF AFFECTIVE DISORDERS	6.533	2013	671	1	Canada	2020
55	Grifoni A. et al.	CELL	66.85	2006	668.67	5	USA	2020
56	Shang J. et al.	NATURE	69.504	2005	668.33	5	USA	2020
57	Chu D.K. et al.	LANCET	202.731	1995	665	1	Canada	2020
58	Shi H. et al.	LANCET INFECTIOUS DISEASES	71.421	1983	661	3	China	2020
59	Jin Z. et al.	NATURE	69.504	1970	656.67	5	China	2020
60	Wang Y. et al.	LANCET	202.731	1961	653.67	2	China	2020
61	To K.K.W. et al.	LANCET INFECTIOUS DISEASES	71.421	1949	649.67	3	Hong Kong	2020
62	Ahorsu D. K. et al.	INTERNATIONAL JOURNAL OF MENTAL HEALTH AND ADDICTION	11.555	1866	1866	3	Iran	2022
63	Verity R. et al.	SCIENCE	63.714	1809	603	3	USA	2020
64	Li R. et al.	LANCET INFECTIOUS DISEASES	71.421	1810	603.33	5	UK	2020
65	Lu X. et al.	NATURE COMMUNICATIONS	17.694	1799	599.67	3	China	2020
66	Ou X. et al.	NEW ENGLAND JOURNAL OF MEDICINE	176.079	1799	599.67	5	China	2020
67	Xu X. W. et al.	BMJ-BRITISH MEDICAL JOURNAL	93.333	1780	593.33	4	China	2020
68	Huang C. et al.	LANCET	202.731	1779	889.5	3	China	2021
69	Kampf G. et al.	JOURNAL OF HOSPITAL INFECTION	8.944	1778	592.67	5	Germany	2020
70	Wolfel R. et al.	NATURE	69.504	1777	592.33	4	Germany	2020
71	He X. et al.	NATURE MEDICINE	87.241	1767	589	5	China	2020
72	Huang Y and Zhao N	PSYCHIATRY RESEARCH	11.225	1763	587.67	4	China	2020
73	Grasselli G. et al.	JAMA-JOURNAL OF THE AMERICAN MEDICAL ASSOCIATION	157.335	1748	582.67	4	Italy	2020
74	Chinazzi M. et al.	SCIENCE	63.714	1742	580.67	5	USA	2020
75	Hu B. et al.	NATURE REVIEWS MICROBIOLOGY	78.297	1739	869.5	5	China	2021
76	Fang Y. et al.	RADIOLOGY	29.146	1725	575	3	China	2020
77	Bikdeli B. et al.	JOURNAL OF THE AMERICAN COLLEGE OF CARDIOLOGY	27.206	1720	573.33	5	USA	2020
78	Jackson L.A. et al.	NEW ENGLAND JOURNAL OF MEDICINE	157.335	1715	571.67	5	USA	2020
79	Rajkumar R. P	ASIAN JOURNAL OF PSYCHIATRY	13.89	1711	570.33	5	India	2020
80	Zhang L. et al.	SCIENCE	63.714	1695	565	5	Germany	2020
81	Shang J. et al.	PROCEEDINGS OF THE NATIONAL ACADEMY OF SCIENCES OF THE UNITED STATES OF AMERICA	12.779	1657	552.33	5	USA	2020
82	Wang Q. et al.	CELL	66.85	1655	572.67	5	China	2020
83	Pappa S. et al.	BRAIN BEHAVIOR AND IMMUNITY	19.227	1654	564	1	UK	2020
84	Grein J. et al.	NEW ENGLAND JOURNAL OF MEDICINE	176.079	1615	559.33	3	UK	2020
85	Gordon D.E. et al.	NATURE	69.504	1612	544.33	5	USA	2020
86	Xiao F. et al.	GASTROENTEROLOGY	33.883	1608	536	5	China	2020
87	Chan J.F.W. et al.	EMERGING MICROBES & INFECTIONS	19.568	1597	532.33	5	China	2020
88	Zhang J. et al.	ALLERGY	14.71	1592	530.67	3	China	2020
89	Docherty A.B. et al.	BMJ-BRITISH MEDICAL JOURNAL	93.333	1585	528.33	3	UK	2020
90	Emanuel E.J. et al.	NEW ENGLAND JOURNAL OF MEDICINE	176.079	1578	526	5	USA	2020
91	Ye Q. et al.	JOURNAL OF INFECTION	38.637	1576	525.33	5	China	2020
92	Sanders J.M. et al.	JAMA-JOURNAL OF THE AMERICAN MEDICAL ASSOCIATION	157.335	1568	525.33	5	USA	2020
93	Helms J. et al.	INTENSIVE CARE MEDICINE	41.787	1565	521.67	3	France	2020
94	Bhatraju P.K. et al.	NEW ENGLAND JOURNAL OF MEDICINE	176.079	1558	519.33	4	USA	2020
95	Khoury D.S. et al.	NATURE MEDICINE	87.241	1548	774	5	Australia	2021
96	Flaxman S. et al.	NATURE	69.504	1538	512.67	5	UK	2020
97	Lopez Bemal J.	NEW ENGLAND JOURNAL OF MEDICINE	176.079	1528	764	4	UK	2021
98	Cheng Y. et al.	KIDNEY INTERNATIONAL	18.998	1525	508.33	3	China	2020
99	Shereen M.A. et al.	JOURNAL OF ADVANCED RESEARCH	12.822	1511	503.67	5	China	2020
100	Singhal T. et al.	INDIAN JOURNAL OF PEDIATRICS	5.319	1508	502.67	5	India	2020

UK: United Kingdom; USA: United States of America; China: China.

**Figure 2.**
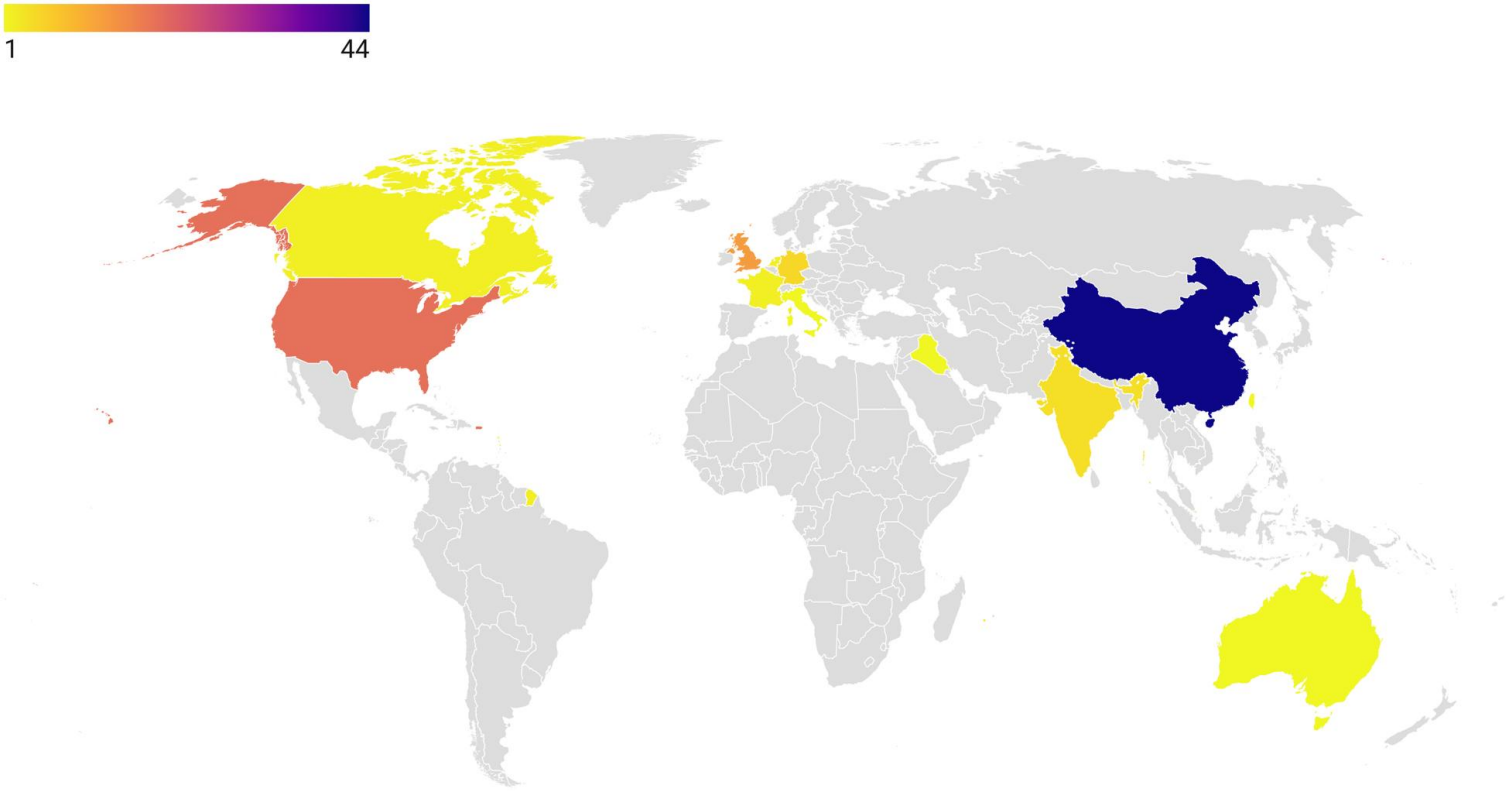
Choropleth map showing the countries contributing to the 100 most-cited articles (dark blue represents high-density of publications, while yellow represents low-density of publications).

Understandably that most of the articles were published in 2020 at the initial phase of the pandemic measuring up to 91 articles. Seven articles were published in 2021, while two articles were noted to be published in 2022. The 100 most-cited articles vary widely in terms of their locations with China producing the most papers (*n* = 44) followed by the USA (*n* = 19) and UK (*n* = 13). Three papers were from an international collaboration, namely, ‘The species Severe acute respiratory syndrome-related coronavirus: classifying 2019-nCoV and naming it SARS-CoV-2’ by Coronaviridae Study Group of the International Committee on Taxonomy of Viruses [8], ‘Clinical management of severe acute respiratory infection (SARI) when COVID-19 disease is suspected. Interim guidance’ by World Health Organization (WHO) [9], and the effectiveness of ADZ1222 vaccine by Voysey [10]. The geographical spread of the 100 most-cited publications was very diversified as demonstrated by Fig. 2, which includes Germany (*n* = 5), India (*n* = 4), Canada (*n* = 2), France (*n* = 2), Netherlands (*n* = 2), Hong Kong (*n* = 1), Italy (*n* = 1), Taiwan (*n* = 1), Iraq (*n* = 1), Singapore (*n* = 1), and Australia (*n* = 1).

**Figure 3.**
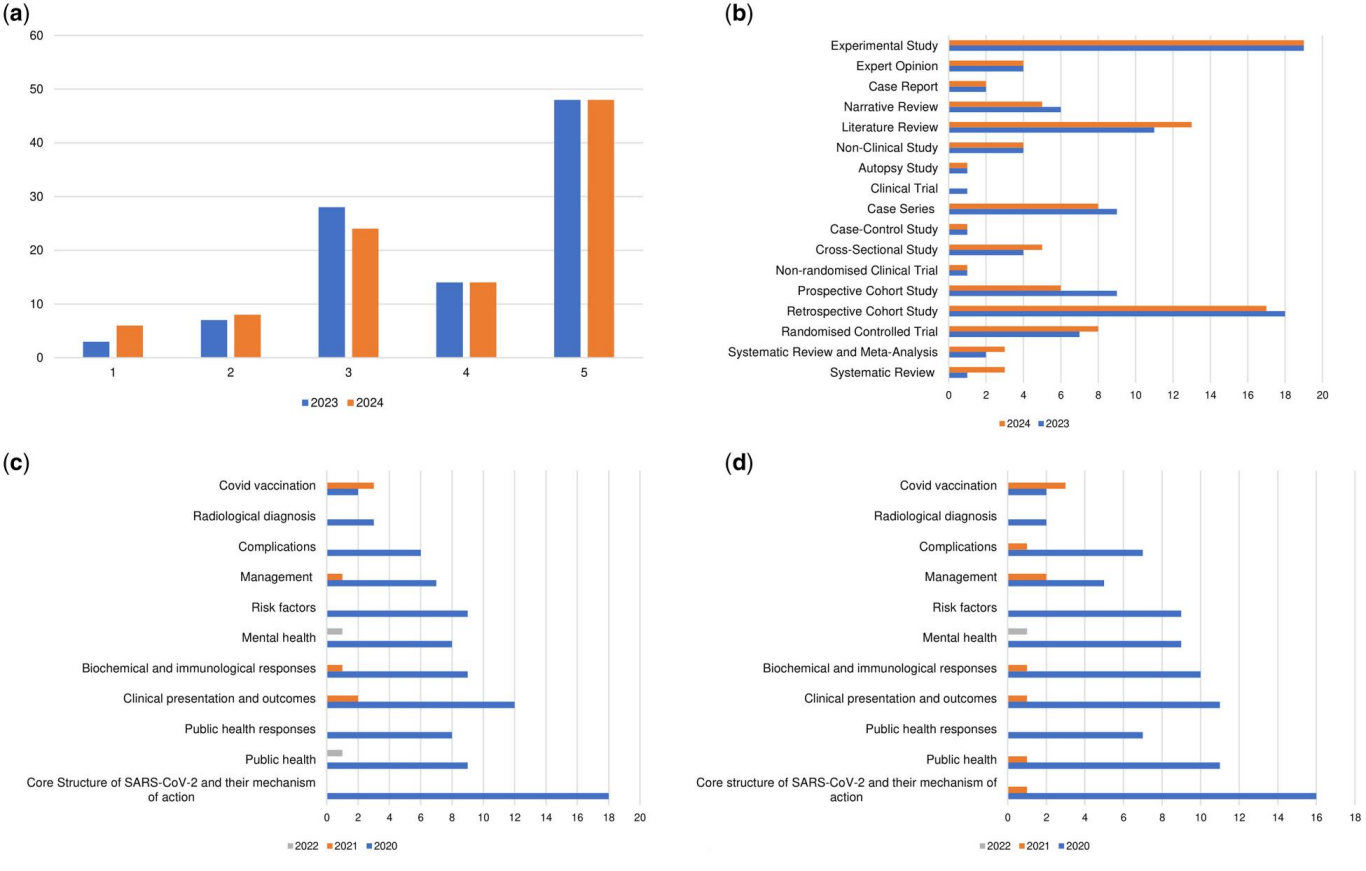
(a) Number of articles based on the level of evidence as per Oxford Centre of Evidence-Based Medicine (OCEBM) classification for both 2023 and 2024 searches. (b) The study designs of the 100 most-cited articles for both 2023 and 2024 searches. (c) Main subjects of the 100 most-cited articles in 2023 (category based on years of publication). (d) Main subjects of the 100 most-cited articles in 2024 (category based on years of publication).

The majority of these articles were of evidence level 5 (*n* = 48) as per Oxford Centre of Evidence-Based Medicine (OCEBM) (Fig. 3a). This was followed by evidence level 3 (*n* = 28) which was predominantly cohort studies (*n* = 24) and one non-labelled non-randomized clinical trial, with retrospective cohort studies contributing 18 articles while prospective contributed nine articles. One of the level 3 papers was an open-labelled non-randomized trial evaluating hydroxychloroquine and azithromycin [11] as a treatment. Fourteen articles were noted to be of level 4 evidence, which includes a mix of case series (*n* = 9), cross-sectional study (*n* = 4), and case-control study (*n* = 1). Level 2 and level 1 evidence-based articles were seven and three papers, respectively. All of the level 2 papers were randomized controlled trials. Two of the level 1 evidence-based articles were systematic reviews and meta-analyses, while the remaining paper was a systematic review assessing the impact of COVID-19 on the general public’s mental health.

A mixture of different studies was noted among the level 5 evidence articles. This includes experimental studies (*n* = 19), case reports (*n* = 2), expert opinion (*n* = 4), narrative review (*n* = 6), literature review (*n* = 11), autopsy study (*n* = 1), non-clinical study (*n* = 4), and clinical trial (*n* = 1) (Fig. 3b).

The main subjects of the articles were well spread across a few categories. Papers assessing the core structure of the SARS-CoV-2 virus and their mechanism of action (*n* = 18), as well as papers assessing the effect of COVID-19 on public health and public health response (*n* = 18) represent the largest number. This is followed by papers investigating the clinical presentation and outcomes, representing 14 publications. Other main subjects of note in descending order were biochemical and immunological responses to COVID-19 infection (*n* = 10), mental health (*n* = 9), risk factors associated with an increased mortality rate (*n* = 9), management of COVID-19 infection (*n* = 8), complications of COVID-19 infection (*n* = 6), and radiological diagnosis of COVID-19 infection (*n* = 3) (Fig. 3c). The common themes for complications of COVID-19 investigated were neurological events (*n* = 1), cardiovascular events (*n* = 2), and thrombotic events (*n* = 3).

Majority of these papers were published in New England Journal of Medicine (*n* = 13), followed by Nature (*n* = 7), and the third most frequently noted journal is shared between Cell (*n* = 6) and Lancet (*n* = 6). The impact factors for each of these journals in 2022 were 176.1, 69.5, 66.9, and 202.7, respectively. The journal with the highest impact factor noted in our study was Lancet (202.7), and the journal with the lowest impact factor found was Pediatria I Medycyna Rodzinna – Paediatrics and Family Medicine (0.1). The published journals for each article with their corresponding impact factors (as of year 2022) can be found in Table 1. Seventy-two papers (72%) were found to have fundings from external sources. Of this, 44 (61.1%) disclosed no specific conflicting of interests. From the 28 papers that disclosed conflict of interests, 2 articles were documented to have received support from the funders outside of the published papers, while 1 article received cash and stocks compensation.

### Comparison search in 2024

The following search carried out in April 2024 gathered a total of 528 745 articles from Web of Science associated with the theme ‘Covid-19’. These were ranked from highest to lowest based on the total number of citations (Supplementary Appendix S2). Collectively, the 100 most-cited COVID-19 articles in 2024 were cited by a total of 316 817 articles.

When compared to the 100 most-cited articles searched in 2023, one article was retracted due to concerns from the publisher, and 11 articles were replaced with different articles of higher citations. Seven articles remained unchanged in terms of ranking, 35 articles had their ranking downgraded, while 47 articles had their ranking upgraded. The highest most-cited article was Zhou et al. with a total citation of 16 610 (previously ranked two in 2023). While the article with the lowest citation in our list belongs to Wynants et al., entitled ‘Prediction models for diagnosis and prognosis of COVID-19 infection: systematic review and critical appraisal’. Figure 3b shows the study design for both 2023 and 2024 searches.

Of the 11 new articles added, 3 were of level 1 evidence, 1 was level 2 evidence, 1 was level 3 evidence, 1 was level 4 evidence, and 5 were level 5 evidence (Fig. 3a). Three of these papers discussed about the public health aspect of the pandemic, which includes surveillances, prediction model, and global pandemic policies. Two of these papers discussed the complications associated with COVID-19 infection focusing on post-COVID syndrome and multisystem inflammatory syndrome in children, whereas one of the articles was a systematic review discussing the impact of COVID-19 infection on mental health. Other themes of discussion were risk factors (*n* = 1), mechanism of action and structure (*n* = 1), biochemical and immunological response (*n* = 1), clinical outcomes (*n* = 1), and management (*n* = 1). Four these new articles were published in 2021, whereas seven of these articles were published in 2020 (Fig. 3d).

Of 47 articles that had their rankings upgraded compared to the list in 2023, majority of these articles were related to mental health (*n* = 8, 88.9% of the previous mental health articles in 2023) followed by COVID vaccination (*n* = 4, 80% of the previous COVID vaccination articles in 2023) and public health responses (*n* = 5, 62.5% of the previous public health responses articles in 2023). Supplementary Appendix S3 tabulates the 100 most-cited articles searched in 2024, giving an overview of their current ranking versus their previous ranking, with newly added articles marked as ‘NEW’.

## Discussion

This study aims to evaluate and summarize the COVID-19 research thus far, to provide insight into the evolution of research focus since the initial phase of the pandemic. This paper also aims to delineate the epidemiology of the 100 most-cited articles and to shed light upon less-covered themes.

Given the timeline of the COVID-19 pandemic, most of the research publications were groundbreaking to the scientific community in 2020, thus resulting in the rapid growth of COVID-19 related scientific studies during the initial phase of the pandemic. This would explain the abundance of publications in 2020 measuring up to 91 articles in our study. At the beginning of the pandemic, there remained an urgent demand to understand the viral structure and mechanism, pathology, clinical presentations, risk factors associated with poor prognosis, and diagnostic evaluation associated with COVID-19 infection, representing a total of 54 articles included in our study. Hence, most of these articles were published in 2020 (*n* = 51). Only three papers were published in 2021 with one of the articles describing the 6-months consequences of patients discharged following a COVID-19 infection [12]. This is in keeping with the progression of pandemic as long-term sequala of COVID-19 infection were starting to be recognized [13].

Summarizing the main subjects found, this study observed a comprehensive breadth of themes around COVID-19. From the 100 most-cited papers gathered, these studies covered management, impact on mental health, biochemical and radiological diagnosis, clinical presentations, clinical outcomes, complications, risk factors, origin, transmission route, core viral structures and genomes, the impact of COVID-19 pandemic on the environment, and COVID-19 vaccination. As mentioned above, there was a clear theme with papers published in 2020, but the same applies to papers published in 2021 and 2022. In 2021, more articles were observed to be investigating the different drug therapies available and also COVID vaccination. The remaining two papers in 2022 assessed the environmental impact of COVID-19 lockdown on the spatial distribution of aerosol [14], and fear of COVID-19 [15]. This has shown that there is a move in terms of research demands to assess prevention, management of acute COVID-19 and long COVID-19 as well as the impact of COVID-19 lockdown on general public and the environment.

Mental health emerged as a significant subject within the literature, with nine articles in 2023 dedicated to this topic. Out of the nine articles discussing mental health, two papers were observed to focus their participants on public health workers, five papers focused on the general public, one paper focused on college students, and one paper provided a panel expert opinion on the research scope. In terms of health workers, the studies collectively found that female nurses and healthcare professionals were at high risk for anxiety, insomnia, and depression. The five articles on general public’s mental health have confirmed the level of distress from COVID-19 pandemic and lockdown as well as risk factors and protective factors against mental health illness. Ahorsu [16] presents a development of a new scale which they termed ‘Fear of Covid-19 Scale’ (FC-19S). This was created following a rigorous literature review to create the first known psychometric assessment. This is the only patient-reported outcome measurement (PROM) described in the current study, which can be utilized by any governing body when planning holistic goals for a COVID-19 fear-free public.

The global scale of COVID-19 pandemic has also shown how the scientific community were able to collaborate both nationally and internationally to bring about advances in medical knowledge and innovative treatments. Zyoud and Al-Jabi [15] demonstrated in their study that China, the USA, UK, and Italy were the leading contributors in early 2020. This was attributed to a high density of COVID-19 infection in the abovementioned countries. The findings were replicated in our study as well whereby most of the current articles were produced by China, the USA, UK, and Europe (in this study, represented by Germany, Italy, Netherlands, and France).

Of the 100 articles searched in 2023, 26 publications were multi-centre collaborations. Three of the 26 articles were of international collaboration, respectively describing the discussion of SARS-CoV-2 virus naming [8], guidelines on management and triaging of COVID-19 infection [9], and the effectiveness of the AZD1222 vaccine [10]. China produces the most national multi-centre collaborative publications totalling 10 papers, followed by UK (*n* = 5) and USA (*n* = 4). Other countries noted in our studies are France (*n* = 1), Hong Kong (*n* = 1), Italy (*n* = 1), and Netherlands (*n* = 1). The majority of these multi-centre articles investigate the clinical presentation and outcomes of COVID-19 infection (*n* = 5), and risk factors associated with poor prognosis (*n* = 5). This is followed by a discussion on management (*n* = 4), COVID-19 vaccination (*n* = 3), mental health (*n* = 3), complications (*n* = 2), biochemical and immunological responses (*n* = 2), and public health (*n* = 1). Concerning management as a main subject, all four articles were multi-centred randomized controlled trials investigating dexamethasone [17], remdesivir [18, 19], and lopinavir-ritonavir [20]. It is not unexpected that the large-scale RECOVERY randomized controlled trial investigating the effect of dexamethasone on mortality rate falls within the 10 most-cited articles. These large-scale randomized controlled trials are fundamental to a pandemic in exploring the necessary interventional measures. A meta-analysis carried out by Dechartres *et al*. [21] have demonstrated that results from smaller-scale studies should be interpreted with caution as the impact of biases were higher.

This study has also shown that accelerated research was achievable given how multiple randomized controlled trials were approved and completed within a short period. Despite so, a systematic review carried out by Kudhail *et al*. [22] in 2022 demonstrated that these accelerated publications were shown to be at risk of bias. This, in particular, is due to the unblinded or single-blinded nature of some studies. Five out of seven randomized controlled trials included in our study are either observer blinded (*n* = 2) or single-blinded (*n* = 3), thus resulting in a higher risk of randomization sampling biases [23].

Subsequent search in 2024 showed more focus on the complications of COVID-19 infection as well as how the appropriate bodies can further reduce transmission risk through non-pharmacological interventions. This is notable based on the themes of the 11 newly added articles to the 100 most-cited list. Furthermore, it is reassuring to note that the impact of COVID-19 and lockdown on mental health is taken seriously by the scientific community.

The main limitations of this study include those that are inherent to most bibliometric analyses. Given that this study only utilizes ‘Web of science’ as the only source of articles, the breadth of publications included will be limited to the abovementioned database only. Second, bibliometric analysis is inherently a quantitative analysis that relies on the citation counts to decide the impact of an article. The underlying assumption drawn in such scenario would be the total number of citations of an article equal to the confidence level of other authors for this publication. This assumption ignores the possibility of authors citing the specific paper for their study design, or facts within their introduction [24]. This study has the added benefit of looking at the changes to the 100 most-cited list by assessing the articles one year apart. Unfortunately, newer studies may be missed as they were not given sufficient time to gather citation counts.

Regardless of the abovementioned limitations, this study presented an extensive literature search into the 100 most-cited literature for COVID-19 while tracking for the stability of these articles one year down the line. The study analysed the current available articles and categorized them based on the time, level of evidence, study design, location of publication, and the study design. This study presented the temporal trend in emerging COVID-19 topics such as the impact of COVID-19 pandemic and lockdown on the environment, mental health, and long COVID-19 syndrome. All themes are the lingering impact of COVID-19.

## Conclusion

The majority of articles included in this study fell within the range of level 3 to level 5 evidence, primarily consisting of experimental studies, literature reviews, case series, and cohort studies. Fortunately, the secondary searched in 2024 showed an increase in number of level 1 and 2 studies compared to 2023, which showed the move from a scientific community to collate stronger evidences. A few noteworthy high-level studies (level 1 and level 2) focused on new management strategies for COVID-19, the impact of the pandemic on the general public’s mental health, and preventive measures to reduce transmission rates. This suggests a shift in research focus within the scientific community as we transition from the peak of the COVID-19 pandemic to a post-pandemic period, emphasizing the need to understand the long-term consequences of the pandemic and lockdown on the general population. Of note is the increasing focus on impact of COVID-19 and lockdown on mental health. It is worth noting that most of the randomized controlled trials in our study were either open-labelled or single-blinded, highlighting the potential for future studies to strive for double-blinded randomized controlled trials to validate the existing findings.

## Supplementary Material

ckae098_Supplementary_Data

## Data Availability

All finalized data and materials generated or analysed during this study are included in this published article. References used in our manuscript can be found in the reference section. The 100 most-cited papers can be found in the appendix, which includes their authors’ names, full title, journals published, date of publication, and DOI (Supplementary material). The raw data are available (including other papers that we have excluded) are available from the corresponding author on reasonable request.
