# Supplementary material for: The 100 most-cited articles in COVID-19: a bibliometric analysis
Source: Eur J Public Health. 2024 Jul 6;34(4):744–52. doi: 10.1093/eurpub/ckae098 (PMC11293834; doi:10.1093/eurpub/ckae098)
Supplement: ckae098_Supplementary_Data [file ckae098_supplementary_data.zip › ckae098_Supplementary_Data/ejph-2023-10-om-0567-File006.docx]

**Appendix 1**

1. Guan W, Ni Z, Hu Y, Liang W, Ou C, He J, et al. Clinical characteristics of Coronavirus Disease 2019 in China. New England Journal of Medicine. 2020;382(18):1708–20. doi:10.1056/nejmoa2002032
2. Zhou F, Yu T, Du R, Fan G, Liu Y, Liu Z, et al. Clinical course and risk factors for mortality of adult inpatients with covid-19 in Wuhan, China: A retrospective cohort study. The Lancet. 2020;395(10229):1054–62. doi:10.1016/s0140-6736(20)30566-3
3. Hoffmann M, Kleine-Weber H, Schroeder S, Krüger N, Herrler T, Erichsen S, et al. SARS-COV-2 cell entry depends on ACE2 and TMPRSS2 and is blocked by a clinically proven protease inhibitor. Cell. 2020;181(2). doi:10.1016/j.cell.2020.02.052
4. Polack FP, Thomas SJ, Kitchin N, Absalon J, Gurtman A, Lockhart S, et al. Safety and efficacy of the BNT162B2 mrna covid-19 vaccine. New England Journal of Medicine. 2020;383(27):2603–15. doi:10.1056/nejmoa2034577
5. Long Q-X, Liu B-Z, Deng H-J, Wu G-C, Deng K, Chen Y-K, et al. Antibody responses to SARS-COV-2 in patients with covid-19. Nature Medicine. 2020;26(6):845–8. doi:10.1038/s41591-020-0897-1
6. The RECOVERY Collaborative Group. Dexamethasone in hospitalized patients with covid-19. New England Journal of Medicine. 2021;384(8):693–704. doi:10.1056/nejmoa2021436
7. Bai Y, Yao L, Wei T, Tian F, Jin D-Y, Chen L, et al. Presumed asymptomatic carrier transmission of COVID-19. JAMA. 2020;323(14):1406. doi:10.1001/jama.2020.2565
8. Mao L, Jin H, Wang M, Hu Y, Chen S, He Q, et al. Neurologic manifestations of hospitalized patients with coronavirus disease 2019 in Wuhan, China. JAMA Neurology. 2020;77(6):683. doi:10.1001/jamaneurol.2020.1127
9. Walls AC, Park Y-J, Tortorici MA, Wall A, McGuire AT, Veesler D. Structure, function, and antigenicity of the SARS-COV-2 spike glycoprotein. Cell. 2020;181(2). doi:10.1016/j.cell.2020.02.058
10. Baden LR, El Sahly HM, Essink B, Kotloff K, Frey S, Novak R, et al. Efficacy and safety of the mrna-1273 SARS-COV-2 vaccine. New England Journal of Medicine. 2021;384(5):403–16. doi:10.1056/nejmoa2035389
11. Wu C, Chen X, Cai Y, Xia J, Zhou X, Xu S, et al. Risk factors associated with acute respiratory distress syndrome and death in patients with coronavirus disease 2019 pneumonia in Wuhan, China. JAMA Internal Medicine. 2020;180(7):934. doi:10.1001/jamainternmed.2020.0994
12. Gorbalenya AE, Baker SC, Baric RS, de Groot RJ, Drosten C, Gulyaeva AA, et al. The species severe acute respiratory syndrome-related coronavirus: Classifying 2019-ncov and naming it SARS-COV-2. Nature Microbiology. 2020;5(4):536–44. doi:10.1038/s41564-020-0695-z
13. Yang X, Yu Y, Xu J, Shu H, Xia J, Liu H, et al. Clinical course and outcomes of critically ill patients with SARS-COV-2 pneumonia in Wuhan, China: A single-centered, retrospective, Observational Study. The Lancet Respiratory Medicine. 2020;8(5):475–81. doi:10.1016/s2213-2600(20)30079-5
14. Beigel JH, Tomashek KM, Dodd LE, Mehta AK, Zingman BS, Kalil AC, et al. Remdesivir for the treatment of covid-19 — final report. New England Journal of Medicine. 2020;383(19):1813–26. doi:10.1056/nejmoa2007764
15. Richardson S, Hirsch JS, Narasimhan M, Crawford JM, McGinn T, Davidson KW, et al. Presenting characteristics, comorbidities, and outcomes among 5700 patients hospitalized with covid-19 in the New York City area. JAMA. 2020;323(20):2052. doi:10.1001/jama.2020.6775
16. Lai J, Ma S, Wang Y, Cai Z, Hu J, Wei N, et al. Factors associated with mental health outcomes among health care workers exposed to coronavirus disease 2019. JAMA Network Open. 2020;3(3). doi:10.1001/jamanetworkopen.2020.3976
17. Klok FA, Kruip MJHA, van der Meer NJM, Arbous MS, Gommers DAMPJ, Kant KM, et al. Incidence of thrombotic complications in critically ill ICU patients with covid-19. Thrombosis Research. 2020;191:145–7. doi:10.1016/j.thromres.2020.04.013
18. Wang C, Pan R, Wan X, Tan Y, Xu L, Ho CS, et al. Immediate psychological responses and associated factors during the initial stage of the 2019 coronavirus disease (covid-19) epidemic among the general population in China. International Journal of Environmental Research and Public Health. 2020;17(5):1729. doi:10.3390/ijerph17051729
19. Ai T, Yang Z, Hou H, Zhan C, Chen C, Lv W, et al. Correlation of chest CT and RT-PCR testing for coronavirus disease 2019 (covid-19) in China: A report of 1014 cases. Radiology. 2020;296(2). doi:10.1148/radiol.2020200642
20. Rani S, Kumar R. Spatial distribution of aerosol optical depth over India during COVID-19 lockdown phase-1. Spatial Information Research. 2022;30(3):417–26. doi:10.1007/s41324-022-00442-9
21. Ruan Q, Yang K, Wang W, Jiang L, Song J. Clinical predictors of mortality due to COVID-19 based on an analysis of data of 150 patients from Wuhan, China. Intensive Care Medicine. 2020;46(5):846–8. doi:10.1007/s00134-020-05991-x
22. Cao B, Wang Y, Wen D, Liu W, Wang J, Fan G, et al. A trial of lopinavir–ritonavir in adults hospitalized with severe covid-19. New England Journal of Medicine. 2020;382(19):1787–99. doi:10.1056/nejmoa2001282
23. Xu Z, Shi L, Wang Y, Zhang J, Huang L, Zhang C, et al. Pathological findings of COVID-19 associated with acute respiratory distress syndrome. The Lancet Respiratory Medicine. 2020;8(4):420–2. doi:10.1016/s2213-2600(20)30076-x
24. Lauer SA, Grantz KH, Bi Q, Jones FK, Zheng Q, Meredith HR, et al. The incubation period of Coronavirus Disease 2019 (COVID-19) from publicly reported confirmed cases: Estimation and application. Annals of Internal Medicine. 2020;172(9):577–82. doi:10.7326/m20-0504
25. Lan J, Ge J, Yu J, Shan S, Zhou H, Fan S, et al. Structure of the SARS-COV-2 spike receptor-binding domain bound to the ACE2 receptor. Nature. 2020;581(7807):215–20. doi:10.1038/s41586-020-2180-5
26. Gautret P, Lagier J-C, Parola P, Hoang VT, Meddeb L, Mailhe M, et al. Hydroxychloroquine and azithromycin as a treatment of COVID-19: Results of an open-label non-randomized clinical trial. International Journal of Antimicrobial Agents. 2020;56(1):105949. doi:10.1016/j.ijantimicag.2020.105949
27. Ackermann M, Verleden SE, Kuehnel M, Haverich A, Welte T, Laenger F, et al. Pulmonary vascular endothelialitis, thrombosis, and angiogenesis in covid-19. New England Journal of Medicine. 2020;383(2):120–8. doi:10.1056/nejmoa2015432
28. Williamson EJ, Walker AJ, Bhaskaran K, Bacon S, Bates C, Morton CE, et al. Factors associated with covid-19-related death using OpenSAFELY. Nature. 2020;584(7821):430–6. doi:10.1038/s41586-020-2521-4
29. Qin C, Zhou L, Hu Z, Zhang S, Yang S, Tao Y, et al. Dysregulation of immune response in patients with coronavirus 2019 (COVID-19) in Wuhan, China. Clinical Infectious Diseases. 2020;71(15):762–8. doi:10.1093/cid/ciaa248
30. Yan R, Zhang Y, Li Y, Xia L, Guo Y, Zhou Q. Structural basis for the recognition of SARS-COV-2 by full-length human ACE2. Science. 2020;367(6485):1444–8. doi:10.1126/science.abb2762
31. Holmes EA, O’Connor RC, Perry VH, Tracey I, Wessely S, Arseneault L, et al. Multidisciplinary research priorities for the COVID-19 pandemic: A call for action for mental health science. The Lancet Psychiatry. 2020;7(6):547–60. doi:10.1016/s2215-0366(20)30168-1
32. Liang W, Guan W, Chen R, Wang W, Li J, Xu K, et al. Cancer patients in SARS-COV-2 infection: A nationwide analysis in China. The Lancet Oncology. 2020;21(3):335–7. doi:10.1016/s1470-2045(20)30096-6
33. Nicola M, Alsafi Z, Sohrabi C, Kerwan A, Al-Jabir A, Iosifidis C, et al. The socio-economic implications of the coronavirus pandemic (COVID-19): A Review. International Journal of Surgery. 2020;78:185–93. doi:10.1016/j.ijsu.2020.04.018
34. Chen G, Wu D, Guo W, Cao Y, Huang D, Wang H, et al. Clinical and immunological features of severe and moderate coronavirus disease 2019. Journal of Clinical Investigation. 2020;130(5):2620–9. doi:10.1172/jci137244
35. Lai C-C, Shih T-P, Ko W-C, Tang H-J, Hsueh P-R. Severe acute respiratory syndrome coronavirus 2 (SARS-COV-2) and coronavirus disease-2019 (COVID-19): The epidemic and the challenges. International Journal of Antimicrobial Agents. 2020;55(3):105924. doi:10.1016/j.ijantimicag.2020.105924
36. Sohrabi C, Alsafi Z, O’Neill N, Khan M, Kerwan A, Al-Jabir A, et al. World Health Organization declares global emergency: A review of the 2019 novel coronavirus (COVID-19). International Journal of Surgery. 2020;76:71–6. doi:10.1016/j.ijsu.2020.02.034
37. Guan W, Liang W, Zhao Y, Liang H, Chen Z, Li Y, et al. Comorbidity and its impact on 1590 patients with covid-19 in China: A nationwide analysis. European Respiratory Journal. 2020;55(5):2000547. doi:10.1183/13993003.00547-2020
38. Dong Y, Mo X, Hu Y, Qi X, Jiang F, Jiang Z, et al. Epidemiology of covid-19 among children in China. Pediatrics. 2020;145(6). doi:10.1542/peds.2020-0702
39. Rothan HA, Byrareddy SN. The epidemiology and pathogenesis of coronavirus disease (COVID-19) outbreak. Journal of Autoimmunity. 2020;109:102433. doi:10.1016/j.jaut.2020.102433
40. World Health Organization. Clinical management of severe acute respiratory infection (SARI) when covid-19 disease is suspected. interim guidance. Pediatria i Medycyna Rodzinna. 2020;16(1):9–26. doi:10.15557/pimr.2020.0003
41. Shi S, Qin M, Shen B, Cai Y, Liu T, Yang F, et al. Association of cardiac injury with mortality in hospitalized patients with COVID-19 in Wuhan, China. JAMA Cardiology. 2020;5(7):802. doi:10.1001/jamacardio.2020.0950
42. Wiersinga WJ, Rhodes A, Cheng AC, Peacock SJ, Prescott HC. Pathophysiology, transmission, diagnosis, and treatment of coronavirus disease 2019 (covid-19). JAMA. 2020;324(8):782. doi:10.1001/jama.2020.12839
43. Chen T, Wu D, Chen H, Yan W, Yang D, Chen G, et al. Clinical characteristics of 113 deceased patients with coronavirus disease 2019: Retrospective Study. BMJ. 2020;m1091. doi:10.1136/bmj.m1091
44. Tay MZ, Poh CM, Rénia L, MacAry PA, Ng LF. The Trinity of COVID-19: Immunity, inflammation and intervention. Nature Reviews Immunology. 2020;20(6):363–74. doi:10.1038/s41577-020-0311-8
45. Guo T, Fan Y, Chen M, Wu X, Zhang L, He T, et al. Cardiovascular implications of fatal outcomes of patients with coronavirus disease 2019 (COVID-19). JAMA Cardiology. 2020;5(7):811. doi:10.1001/jamacardio.2020.1017
46. Cao W, Fang Z, Hou G, Han M, Xu X, Dong J, et al. The psychological impact of the COVID-19 epidemic on college students in China. Psychiatry Research. 2020;287:112934. doi:10.1016/j.psychres.2020.112934
47. Voysey M, Clemens SA, Madhi SA, Weckx LY, Folegatti PM, Aley PK, et al. Safety and efficacy of the chadox1 ncov-19 vaccine (AZD1222) against SARS-COV-2: An interim analysis of four randomised controlled trials in Brazil, South Africa, and the UK. The Lancet. 2021;397(10269):99–111. doi:10.1016/s0140-6736(20)32661-1
48. Guo Y-R, Cao Q-D, Hong Z-S, Tan Y-Y, Chen S-D, Jin H-J, et al. The origin, transmission and clinical therapies on coronavirus disease 2019 (covid-19) outbreak – an update on the status. Military Medical Research. 2020;7(1). doi:10.1186/s40779-020-00240-0
49. Blanco-Melo D, Nilsson-Payant BE, Liu W-C, Uhl S, Hoagland D, Møller R, et al. Imbalanced host response to SARS-COV-2 drives development of COVID-19. Cell. 2020;181(5). doi:10.1016/j.cell.2020.04.026
50. Bavel JJ, Baicker K, Boggio PS, Capraro V, Cichocka A, Cikara M, et al. Using social and behavioural science to support COVID-19 pandemic response. Nature Human Behaviour. 2020;4(5):460–71. doi:10.1038/s41562-020-0884-z
51. Dhama K, Khan S, Tiwari R, Sircar S, Bhat S, Malik YS, et al. Coronavirus disease 2019–COVID-19. Clinical Microbiology Reviews. 2020;33(4). doi:10.1128/cmr.00028-20
52. Korber B, Fischer WM, Gnanakaran S, Yoon H, Theiler J, Abfalterer W, et al. Tracking changes in SARS-COV-2 spike: Evidence that D614g increases infectivity of the COVID-19 virus. Cell. 2020;182(4). doi:10.1016/j.cell.2020.06.043
53. Chen H, Guo J, Wang C, Luo F, Yu X, Zhang W, et al. Clinical characteristics and intrauterine vertical transmission potential of covid-19 infection in nine pregnant women: A retrospective review of medical records. The Lancet. 2020;395(10226):809–15. doi:10.1016/s0140-6736(20)30360-3
54. Xiong J, Lipsitz O, Nasri F, Lui LMW, Gill H, Phan L, et al. Impact of covid-19 pandemic on Mental Health in the general population: A systematic review. Journal of Affective Disorders. 2020;277:55–64. doi:10.1016/j.jad.2020.08.001
55. Grifoni A, Weiskopf D, Ramirez SI, Mateus J, Dan JM, Moderbacher CR, et al. Targets of T cell responses to SARS-COV-2 coronavirus in humans with covid-19 disease and unexposed individuals. Cell. 2020;181(7). doi:10.1016/j.cell.2020.05.015
56. Shang J, Ye G, Shi K, Wan Y, Luo C, Aihara H, et al. Structural basis of receptor recognition by SARS-COV-2. Nature. 2020;581(7807):221–4. doi:10.1038/s41586-020-2179-y
57. Chu DK, Akl EA, Duda S, Solo K, Yaacoub S, Schünemann HJ, et al. Physical distancing, face masks, and eye protection to prevent person-to-person transmission of SARS-COV-2 and COVID-19: A systematic review and meta-analysis. The Lancet. 2020;395(10242):1973–87. doi:10.1016/s0140-6736(20)31142-9
58. Shi H, Han X, Jiang N, Cao Y, Alwalid O, Gu J, et al. Radiological findings from 81 patients with covid-19 pneumonia in Wuhan, China: A descriptive study. The Lancet Infectious Diseases. 2020;20(4):425–34. doi:10.1016/s1473-3099(20)30086-4
59. Jin Z, Du X, Xu Y, Deng Y, Liu M, Zhao Y, et al. Structure of mpro from SARS-COV-2 and discovery of its inhibitors. Nature. 2020;582(7811):289–93. doi:10.1038/s41586-020-2223-y
60. Wang Y, Zhang D, Du G, Du R, Zhao J, Jin Y, et al. Remdesivir in adults with severe COVID-19: A randomised, double-blind, placebo-controlled, multicentre trial. The Lancet. 2020;395(10236):1569–78. doi:10.1016/s0140-6736(20)31022-9
61. To KK-W, Tsang OT-Y, Leung W-S, Tam AR, Wu T-C, Lung DC, et al. Temporal profiles of viral load in posterior oropharyngeal saliva samples and serum antibody responses during infection by SARS-COV-2: An observational cohort study. The Lancet Infectious Diseases. 2020;20(5):565–74. doi:10.1016/s1473-3099(20)30196-1
62. Ahorsu DK, Lin C-Y, Imani V, Saffari M, Griffiths MD, Pakpour AH. The fear of covid-19 scale: Development and initial validation. International Journal of Mental Health and Addiction. 2020;20(3):1537–45. doi:10.1007/s11469-020-00270-8
63. Verity R, Okell LC, Dorigatti I, Winskill P, Whittaker C, Imai N, et al. Estimates of the severity of coronavirus disease 2019: A model-based analysis. The Lancet Infectious Diseases. 2020;20(6):669–77. doi:10.1016/s1473-3099(20)30243-7
64. Li R, Pei S, Chen B, Song Y, Zhang T, Yang W, et al. Substantial undocumented infection facilitates the rapid dissemination of novel coronavirus (SARS-COV-2). Science. 2020;368(6490):489–93. doi:10.1126/science.abb3221
65. Lu X, Zhang L, Du H, Zhang J, Li YY, Qu J, et al. SARS-COV-2 infection in children. New England Journal of Medicine. 2020;382(17):1663–5. doi:10.1056/nejmc2005073
66. Ou X, Liu Y, Lei X, Li P, Mi D, Ren L, et al. Characterization of spike glycoprotein of SARS-COV-2 on virus entry and its immune cross-reactivity with SARS-COV. Nature Communications. 2020;11(1). doi:10.1038/s41467-020-15562-9
67. Xu X-W, Wu X-X, Jiang X-G, Xu K-J, Ying L-J, Ma C-L, et al. Clinical findings in a group of patients infected with the 2019 novel coronavirus (SARS-COV-2) outside of Wuhan, China: Retrospective case series. BMJ. 2020;m606. doi:10.1136/bmj.m606
68. Huang C, Huang L, Wang Y, Li X, Ren L, Gu X, et al. 6-month consequences of COVID-19 in patients discharged from hospital: A cohort study. The Lancet. 2021;397(10270):220–32. doi:10.1016/s0140-6736(20)32656-8
69. Kampf G, Todt D, Pfaender S, Steinmann E. Persistence of coronaviruses on inanimate surfaces and their inactivation with biocidal agents. Journal of Hospital Infection. 2020;104(3):246–51. doi:10.1016/j.jhin.2020.01.022
70. Wölfel R, Corman VM, Guggemos W, Seilmaier M, Zange S, Müller MA, et al. Virological assessment of hospitalized patients with Covid-2019. Nature. 2020;581(7809):465–9. doi:10.1038/s41586-020-2196-x
71. He X, Lau EH, Wu P, Deng X, Wang J, Hao X, et al. Temporal Dynamics in viral shedding and transmissibility of COVID-19. 2020; doi:10.1101/2020.03.15.20036707
72. Huang Y, Zhao N. Generalized anxiety disorder, depressive symptoms and sleep quality during COVID-19 outbreak in China: A web-based cross-sectional survey. Psychiatry Research. 2020;288:112954. doi:10.1016/j.psychres.2020.112954
73. Grasselli G, Zangrillo A, Zanella A, Antonelli M, Cabrini L, Castelli A, et al. Baseline characteristics and outcomes of 1591 patients infected with SARS-COV-2 admitted to icus of the Lombardy region, Italy. JAMA. 2020;323(16):1574. doi:10.1001/jama.2020.5394
74. Chinazzi M, Davis JT, Ajelli M, Gioannini C, Litvinova M, Merler S, et al. The effect of travel restrictions on the spread of the 2019 novel coronavirus (COVID-19) outbreak. Science. 2020;368(6489):395–400. doi:10.1126/science.aba9757
75. Hu B, Guo H, Zhou P, Shi Z-L. Characteristics of SARS-COV-2 and COVID-19. Nature Reviews Microbiology. 2020;19(3):141–54. doi:10.1038/s41579-020-00459-7
76. Fang Y, Zhang H, Xie J, Lin M, Ying L, Pang P, et al. Sensitivity of chest CT for covid-19: Comparison to RT-PCR. Radiology. 2020;296(2). doi:10.1148/radiol.2020200432
77. Bikdeli B, Madhavan MV, Jimenez D, Chuich T, Dreyfus I, Driggin E, et al. Covid-19 and thrombotic or thromboembolic disease: Implications for prevention, antithrombotic therapy, and follow-up. Journal of the American College of Cardiology. 2020;75(23):2950–73. doi:10.1016/j.jacc.2020.04.031
78. Jackson LA, Anderson EJ, Rouphael NG, Roberts PC, Makhene M, Coler RN, et al. An mrna vaccine against SARS-COV-2 — preliminary report. New England Journal of Medicine. 2020;383(20):1920–31. doi:10.1056/nejmoa2022483
79. Rajkumar RP. Covid-19 and mental health: A review of the existing literature. Asian Journal of Psychiatry. 2020;52:102066. doi:10.1016/j.ajp.2020.102066
80. Zhang L, Lin D, Sun X, Curth U, Drosten C, Sauerhering L, et al. Crystal structure of SARS-COV-2 main protease provides a basis for design of improved α-ketoamide inhibitors. Science. 2020;368(6489):409–12. doi:10.1126/science.abb3405
81. Shang J, Wan Y, Luo C, Ye G, Geng Q, Auerbach A, et al. Cell entry mechanisms of SARS-COV-2. Proceedings of the National Academy of Sciences. 2020;117(21):11727–34. doi:10.1073/pnas.2003138117
82. Wang Q, Zhang Y, Wu L, Niu S, Song C, Zhang Z, et al. Structural and functional basis of SARS-COV-2 entry by using human ACE2. Cell. 2020;181(4). doi:10.1016/j.cell.2020.03.045
83. Pappa S, Ntella V, Giannakas T, Giannakoulis VG, Papoutsi E, Katsaounou P. Prevalence of depression, anxiety, and insomnia among healthcare workers during the COVID-19 pandemic: A systematic review and meta-analysis. Brain, Behavior, and Immunity. 2020;88:901–7. doi:10.1016/j.bbi.2020.05.026
84. Grein J, Ohmagari N, Shin D, Diaz G, Asperges E, Castagna A, et al. Compassionate use of Remdesivir for patients with severe COVID-19. New England Journal of Medicine. 2020;382(24):2327–36. doi:10.1056/nejmoa2007016
85. Gordon DE, Jang GM, Bouhaddou M, Xu J, Obernier K, White KM, et al. A SARS-COV-2 protein interaction map reveals targets for drug repurposing. Nature. 2020;583(7816):459–68. doi:10.1038/s41586-020-2286-9
86. Xiao F, Tang M, Zheng X, Liu Y, Li X, Shan H. Evidence for gastrointestinal infection of SARS-COV-2. Gastroenterology. 2020;158(6). doi:10.1053/j.gastro.2020.02.055
87. Chan JF-W, Kok K-H, Zhu Z, Chu H, To KK-W, Yuan S, et al. Genomic characterization of the 2019 novel human-pathogenic coronavirus isolated from a patient with atypical pneumonia after visiting Wuhan. Emerging Microbes & Infections. 2020;9(1):221–36. doi:10.1080/22221751.2020.1719902
88. Zhang J, Dong X, Cao Y, Yuan Y, Yang Y, Yan Y, et al. Clinical characteristics of 140 patients infected with SARS‐COV‐2 in Wuhan, China. Allergy. 2020;75(7):1730–41. doi:10.1111/all.14238
89. Docherty AB, Harrison EM, Green CA, Hardwick HE, Pius R, Norman L, et al. Features of 20133 UK patients in hospital with covid-19 using the ISARIC who clinical characterisation protocol: Prospective observational cohort study. BMJ. 2020;m1985. doi:10.1136/bmj.m1985
90. Emanuel EJ, Persad G, Upshur R, Thome B, Parker M, Glickman A, et al. Fair allocation of scarce medical resources in the time of covid-19. New England Journal of Medicine. 2020;382(21):2049–55. doi:10.1056/nejmsb2005114
91. Ye Q, Wang B, Mao J. The pathogenesis and treatment of the `cytokine storm’ in covid-19. Journal of Infection. 2020;80(6):607–13. doi:10.1016/j.jinf.2020.03.037
92. Sanders JM, Monogue ML, Jodlowski TZ, Cutrell JB. Pharmacologic treatments for coronavirus disease 2019 (COVID-19). JAMA. 2020; doi:10.1001/jama.2020.6019
93. Helms J, Tacquard C, Severac F, Leonard-Lorant I, Ohana M, Delabranche X, et al. High risk of thrombosis in patients with severe SARS-COV-2 infection: A multicenter prospective cohort study. Intensive Care Medicine. 2020;46(6):1089–98. doi:10.1007/s00134-020-06062-x
94. Bhatraju PK, Ghassemieh BJ, Nichols M, Kim R, Jerome KR, Nalla AK, et al. Covid-19 in critically ill patients in the Seattle region — Case series. New England Journal of Medicine. 2020;382(21):2012–22. doi:10.1056/nejmoa2004500
95. Khoury DS, Cromer D, Reynaldi A, Schlub TE, Wheatley AK, Juno JA, et al. Neutralizing antibody levels are highly predictive of immune protection from symptomatic SARS-COV-2 infection. Nature Medicine. 2021;27(7):1205–11. doi:10.1038/s41591-021-01377-8
96. Flaxman S, Mishra S, Gandy A, Unwin HJ, Mellan TA, Coupland H, et al. Estimating the effects of non-pharmaceutical interventions on COVID-19 in Europe. Nature. 2020;584(7820):257–61. doi:10.1038/s41586-020-2405-7
97. Lopez Bernal J, Andrews N, Gower C, Gallagher E, Simmons R, Thelwall S, et al. Effectiveness of covid-19 vaccines against the B.1.617.2 (delta) variant. New England Journal of Medicine. 2021;385(7):585–94. doi:10.1056/nejmoa2108891
98. Cheng Y, Luo R, Wang K, Zhang M, Wang Z, Dong L, et al. Kidney disease is associated with in-hospital death of patients with covid-19. Kidney International. 2020;97(5):829–38. doi:10.1016/j.kint.2020.03.005
99. Shereen MA, Khan S, Kazmi A, Bashir N, Siddique R. Covid-19 infection: Emergence, transmission, and characteristics of human coronaviruses. Journal of Advanced Research. 2020;24:91–8. doi:10.1016/j.jare.2020.03.005
100. Singhal T. A review of Coronavirus Disease-2019 (COVID-19). The Indian Journal of Pediatrics. 2020;87(4):281–6. doi:10.1007/s12098-020-03263-6
