# Supplementary material for: The 100 most-cited articles in COVID-19: a bibliometric analysis
Source: Eur J Public Health. 2024 Jul 6;34(4):744–52. doi: 10.1093/eurpub/ckae098 (PMC11293834; doi:10.1093/eurpub/ckae098)
Supplement: ckae098_Supplementary_Data [file ckae098_supplementary_data.zip › ckae098_Supplementary_Data/ejph-2023-10-om-0567-File007.docx]

**Appendix 2**

1. Zhou F, Yu T, Du R, Fan G, Liu Y, Liu Z, et al. Clinical course and risk factors for mortality of adult inpatients with covid-19 in Wuhan, China: A retrospective cohort study. The Lancet. 2020;395(10229):1054–62. doi:10.1016/s0140-6736(20)30566-3
2. Hoffmann M, Kleine-Weber H, Schroeder S, Krüger N, Herrler T, Erichsen S, et al. SARS-COV-2 cell entry depends on ACE2 and TMPRSS2 and is blocked by a clinically proven protease inhibitor. Cell. 2020;181(2). doi:10.1016/j.cell.2020.02.052
3. Guan W, Ni Z, Hu Y, Liang W, Ou C, He J, et al. Clinical characteristics of Coronavirus Disease 2019 in China. New England Journal of Medicine. 2020;382(18):1708–20. doi:10.1056/nejmoa2002032
4. Polack FP, Thomas SJ, Kitchin N, Absalon J, Gurtman A, Lockhart S, et al. Safety and efficacy of the BNT162B2 mrna covid-19 vaccine. New England Journal of Medicine. 2020;383(27):2603–15. doi:10.1056/nejmoa2034577
5. The RECOVERY Collaborative Group. Dexamethasone in hospitalized patients with covid-19. New England Journal of Medicine. 2021;384(8):693–704. doi:10.1056/nejmoa2021436
6. Baden LR, El Sahly HM, Essink B, Kotloff K, Frey S, Novak R, et al. Efficacy and safety of the mrna-1273 SARS-COV-2 vaccine. New England Journal of Medicine. 2021;384(5):403–16. doi:10.1056/nejmoa2035389
7. Walls AC, Park Y-J, Tortorici MA, Wall A, McGuire AT, Veesler D. Structure, function, and antigenicity of the SARS-COV-2 spike glycoprotein. Cell. 2020;181(2). doi:10.1016/j.cell.2020.02.058
8. Mao L, Jin H, Wang M, Hu Y, Chen S, He Q, et al. Neurologic manifestations of hospitalized patients with coronavirus disease 2019 in Wuhan, China. JAMA Neurology. 2020;77(6):683. doi:10.1001/jamaneurol.2020.1127
9. Gorbalenya AE, Baker SC, Baric RS, de Groot RJ, Drosten C, Gulyaeva AA, et al. The species severe acute respiratory syndrome-related coronavirus: Classifying 2019-ncov and naming it SARS-COV-2. Nature Microbiology. 2020;5(4):536–44. doi:10.1038/s41564-020-0695-z
10. Long Q-X, Liu B-Z, Deng H-J, Wu G-C, Deng K, Chen Y-K, et al. Antibody responses to SARS-COV-2 in patients with covid-19. Nature Medicine. 2020;26(6):845–8. doi:10.1038/s41591-020-0897-1
11. Wu C, Chen X, Cai Y, Xia J, Zhou X, Xu S, et al. Risk factors associated with acute respiratory distress syndrome and death in patients with coronavirus disease 2019 pneumonia in Wuhan, China. JAMA Internal Medicine. 2020;180(7):934. doi:10.1001/jamainternmed.2020.0994
12. Wang C, Pan R, Wan X, Tan Y, Xu L, Ho CS, et al. Immediate psychological responses and associated factors during the initial stage of the 2019 coronavirus disease (covid-19) epidemic among the general population in China. International Journal of Environmental Research and Public Health. 2020;17(5):1729. doi:10.3390/ijerph17051729
13. Yang X, Yu Y, Xu J, Shu H, Xia J, Liu H, et al. Clinical course and outcomes of critically ill patients with SARS-COV-2 pneumonia in Wuhan, China: A single-centered, retrospective, Observational Study. The Lancet Respiratory Medicine. 2020;8(5):475–81. doi:10.1016/s2213-2600(20)30079-5
14. Beigel JH, Tomashek KM, Dodd LE, Mehta AK, Zingman BS, Kalil AC, et al. Remdesivir for the treatment of covid-19 — final report. New England Journal of Medicine. 2020;383(19):1813–26. doi:10.1056/nejmoa2007764
15. Lai J, Ma S, Wang Y, Cai Z, Hu J, Wei N, et al. Factors associated with mental health outcomes among health care workers exposed to coronavirus disease 2019. JAMA Network Open. 2020;3(3). doi:10.1001/jamanetworkopen.2020.3976
16. Bai Y, Yao L, Wei T, Tian F, Jin D-Y, Chen L, et al. Presumed asymptomatic carrier transmission of COVID-19. JAMA. 2020;323(14):1406. doi:10.1001/jama.2020.2565
17. Ruan Q, Yang K, Wang W, Jiang L, Song J. Clinical predictors of mortality due to COVID-19 based on an analysis of data of 150 patients from Wuhan, China. Intensive Care Medicine. 2020;46(5):846–8. doi:10.1007/s00134-020-05991-x
18. Lan J, Ge J, Yu J, Shan S, Zhou H, Fan S, et al. Structure of the SARS-COV-2 spike receptor-binding domain bound to the ACE2 receptor. Nature. 2020;581(7807):215–20. doi:10.1038/s41586-020-2180-5
19. Dhama K, Khan S, Tiwari R, Sircar S, Bhat S, Malik YS, et al. Coronavirus disease 2019–COVID-19. Clinical Microbiology Reviews. 2020;33(4). doi:10.1128/cmr.00028-20
20. Richardson S, Hirsch JS, Narasimhan M, Crawford JM, McGinn T, Davidson KW, et al. Presenting characteristics, comorbidities, and outcomes among 5700 patients hospitalized with covid-19 in the New York City area. JAMA. 2020;323(20):2052. doi:10.1001/jama.2020.677
21. Williamson EJ, Walker AJ, Bhaskaran K, Bacon S, Bates C, Morton CE, et al. Factors associated with covid-19-related death using OpenSAFELY. Nature. 2020;584(7821):430–6. doi:10.1038/s41586-020-2521-4
22. Ackermann M, Verleden SE, Kuehnel M, Haverich A, Welte T, Laenger F, et al. Pulmonary vascular endothelialitis, thrombosis, and angiogenesis in covid-19. New England Journal of Medicine. 2020;383(2):120–8. doi:10.1056/nejmoa2015432
23. Lauer SA, Grantz KH, Bi Q, Jones FK, Zheng Q, Meredith HR, et al. The incubation period of Coronavirus Disease 2019 (COVID-19) from publicly reported confirmed cases: Estimation and application. Annals of Internal Medicine. 2020;172(9):577–82. doi:10.7326/m20-0504
24. Holmes EA, O’Connor RC, Perry VH, Tracey I, Wessely S, Arseneault L, et al. Multidisciplinary research priorities for the COVID-19 pandemic: A call for action for mental health science. The Lancet Psychiatry. 2020;7(6):547–60. doi:10.1016/s2215-0366(20)30168-1
25. Cao B, Wang Y, Wen D, Liu W, Wang J, Fan G, et al. A trial of lopinavir–ritonavir in adults hospitalized with severe covid-19. New England Journal of Medicine. 2020;382(19):1787–99. doi:10.1056/nejmoa2001282
26. Nicola M, Alsafi Z, Sohrabi C, Kerwan A, Al-Jabir A, Iosifidis C, et al. The socio-economic implications of the coronavirus pandemic (COVID-19): A Review. International Journal of Surgery. 2020;78:185–93. doi:10.1016/j.ijsu.2020.04.018
27. Qin C, Zhou L, Hu Z, Zhang S, Yang S, Tao Y, et al. Dysregulation of immune response in patients with coronavirus 2019 (COVID-19) in Wuhan, China. Clinical Infectious Diseases. 2020;71(15):762–8. doi:10.1093/cid/ciaa248
28. Yan R, Zhang Y, Li Y, Xia L, Guo Y, Zhou Q. Structural basis for the recognition of SARS-COV-2 by full-length human ACE2. Science. 2020;367(6485):1444–8. doi:10.1126/science.abb2762
29. Gautret P, Lagier J-C, Parola P, Hoang VT, Meddeb L, Mailhe M, et al. Hydroxychloroquine and azithromycin as a treatment of COVID-19: Results of an open-label non-randomized clinical trial. International Journal of Antimicrobial Agents. 2020;56(1):105949. doi:10.1016/j.ijantimicag.2020.105949
30. Chen G, Wu D, Guo W, Cao Y, Huang D, Wang H, et al. Clinical and immunological features of severe and moderate coronavirus disease 2019. Journal of Clinical Investigation. 2020;130(5):2620–9. doi:10.1172/jci137244
31. Wiersinga WJ, Rhodes A, Cheng AC, Peacock SJ, Prescott HC. Pathophysiology, transmission, diagnosis, and treatment of coronavirus disease 2019 (covid-19). JAMA. 2020;324(8):782. doi:10.1001/jama.2020.12839
32. Sohrabi C, Alsafi Z, O’Neill N, Khan M, Kerwan A, Al-Jabir A, et al. World Health Organization declares global emergency: A review of the 2019 novel coronavirus (COVID-19). International Journal of Surgery. 2020;76:71–6. doi:10.1016/j.ijsu.2020.02.034
33. Liang W, Guan W, Chen R, Wang W, Li J, Xu K, et al. Cancer patients in SARS-COV-2 infection: A nationwide analysis in China. The Lancet Oncology. 2020;21(3):335–7. doi:10.1016/s1470-2045(20)30096-6
34. Voysey M, Clemens SA, Madhi SA, Weckx LY, Folegatti PM, Aley PK, et al. Safety and efficacy of the chadox1 ncov-19 vaccine (AZD1222) against SARS-COV-2: An interim analysis of four randomised controlled trials in Brazil, South Africa, and the UK. The Lancet. 2021;397(10269):99–111. doi:10.1016/s0140-6736(20)32661-1
35. Cao W, Fang Z, Hou G, Han M, Xu X, Dong J, et al. The psychological impact of the COVID-19 epidemic on college students in China. Psychiatry Research. 2020;287:112934. doi:10.1016/j.psychres.2020.112934
36. Xiong J, Lipsitz O, Nasri F, Lui LMW, Gill H, Phan L, et al. Impact of covid-19 pandemic on Mental Health in the general population: A systematic review. Journal of Affective Disorders. 2020;277:55–64. doi:10.1016/j.jad.2020.08.001
37. Rothan HA, Byrareddy SN. The epidemiology and pathogenesis of coronavirus disease (COVID-19) outbreak. Journal of Autoimmunity. 2020;109:102433. doi:10.1016/j.jaut.2020.102433
38. Xu Z, Shi L, Wang Y, Zhang J, Huang L, Zhang C, et al. Pathological findings of COVID-19 associated with acute respiratory distress syndrome. The Lancet Respiratory Medicine. 2020;8(4):420–2. doi:10.1016/s2213-2600(20)30076-x
39. Tay MZ, Poh CM, Rénia L, MacAry PA, Ng LF. The Trinity of COVID-19: Immunity, inflammation and intervention. Nature Reviews Immunology. 2020;20(6):363–74. doi:10.1038/s41577-020-0311-8
40. Bavel JJ, Baicker K, Boggio PS, Capraro V, Cichocka A, Cikara M, et al. Using social and behavioural science to support COVID-19 pandemic response. Nature Human Behaviour. 2020;4(5):460–71. doi:10.1038/s41562-020-0884-z
41. Hu B, Guo H, Zhou P, Shi Z-L. Characteristics of SARS-COV-2 and COVID-19. Nature Reviews Microbiology. 2020;19(3):141–54. doi:10.1038/s41579-020-00459-7
42. Shi S, Qin M, Shen B, Cai Y, Liu T, Yang F, et al. Association of cardiac injury with mortality in hospitalized patients with COVID-19 in Wuhan, China. JAMA Cardiology. 2020;5(7):802. doi:10.1001/jamacardio.2020.0950
43. Blanco-Melo D, Nilsson-Payant BE, Liu W-C, Uhl S, Hoagland D, Møller R, et al. Imbalanced host response to SARS-COV-2 drives development of COVID-19. Cell. 2020;181(5). doi:10.1016/j.cell.2020.04.026
44. World Health Organization. Clinical management of severe acute respiratory infection (SARI) when covid-19 disease is suspected. interim guidance. Pediatria i Medycyna Rodzinna. 2020;16(1):9–26. doi:10.15557/pimr.2020.0003
45. Guo T, Fan Y, Chen M, Wu X, Zhang L, He T, et al. Cardiovascular implications of fatal outcomes of patients with coronavirus disease 2019 (COVID-19). JAMA Cardiology. 2020;5(7):811. doi:10.1001/jamacardio.2020.1017
46. Klok FA, Kruip MJHA, van der Meer NJM, Arbous MS, Gommers DAMPJ, Kant KM, et al. Incidence of thrombotic complications in critically ill ICU patients with covid-19. Thrombosis Research. 2020;191:145–7. doi:10.1016/j.thromres.2020.04.013
47. Lai C-C, Shih T-P, Ko W-C, Tang H-J, Hsueh P-R. Severe acute respiratory syndrome coronavirus 2 (SARS-COV-2) and coronavirus disease-2019 (COVID-19): The epidemic and the challenges. International Journal of Antimicrobial Agents. 2020;55(3):105924. doi:10.1016/j.ijantimicag.2020.105924
48. Chen T, Wu D, Chen H, Yan W, Yang D, Chen G, et al. Clinical characteristics of 113 deceased patients with coronavirus disease 2019: Retrospective Study. BMJ. 2020;m1091. doi:10.1136/bmj.m1091
49. Ai T, Yang Z, Hou H, Zhan C, Chen C, Lv W, et al. Correlation of chest CT and RT-PCR testing for coronavirus disease 2019 (covid-19) in China: A report of 1014 cases. Radiology. 2020;296(2). doi:10.1148/radiol.2020200642
50. Ahorsu DK, Lin C-Y, Imani V, Saffari M, Griffiths MD, Pakpour AH. The fear of covid-19 scale: Development and initial validation. International Journal of Mental Health and Addiction. 2020;20(3):1537–45. doi:10.1007/s11469-020-00270-8
51. Korber B, Fischer WM, Gnanakaran S, Yoon H, Theiler J, Abfalterer W, et al. Tracking changes in SARS-COV-2 spike: Evidence that D614g increases infectivity of the COVID-19 virus. Cell. 2020;182(4). doi:10.1016/j.cell.2020.06.043
52. Guo Y-R, Cao Q-D, Hong Z-S, Tan Y-Y, Chen S-D, Jin H-J, et al. The origin, transmission and clinical therapies on coronavirus disease 2019 (covid-19) outbreak – an update on the status. Military Medical Research. 2020;7(1). doi:10.1186/s40779-020-00240-0
53. Dong Y, Mo X, Hu Y, Qi X, Jiang F, Jiang Z, et al. Epidemiology of covid-19 among children in China. Pediatrics. 2020;145(6). doi:10.1542/peds.2020-0702
54. Grifoni A, Weiskopf D, Ramirez SI, Mateus J, Dan JM, Moderbacher CR, et al. Targets of T cell responses to SARS-COV-2 coronavirus in humans with covid-19 disease and unexposed individuals. Cell. 2020;181(7). doi:10.1016/j.cell.2020.05.015
55. Chu DK, Akl EA, Duda S, Solo K, Yaacoub S, Schünemann HJ, et al. Physical distancing, face masks, and eye protection to prevent person-to-person transmission of SARS-COV-2 and COVID-19: A systematic review and meta-analysis. The Lancet. 2020;395(10242):1973–87. doi:10.1016/s0140-6736(20)31142-9
56. Shang J, Ye G, Shi K, Wan Y, Luo C, Aihara H, et al. Structural basis of receptor recognition by SARS-COV-2. Nature. 2020;581(7807):221–4. doi:10.1038/s41586-020-2179-y
57. Khoury DS, Cromer D, Reynaldi A, Schlub TE, Wheatley AK, Juno JA, et al. Neutralizing antibody levels are highly predictive of immune protection from symptomatic SARS-COV-2 infection. Nature Medicine. 2021;27(7):1205–11. doi:10.1038/s41591-021-01377-8
58. Nalbandian, A., Sehgal, K., Gupta, A. et al. Post-acute COVID-19 syndrome. Nat Med 27, 601–615 (2021). <https://doi.org/10.1038/s41591-021-01283-z>
59. Guan W, Liang W, Zhao Y, Liang H, Chen Z, Li Y, et al. Comorbidity and its impact on 1590 patients with covid-19 in China: A nationwide analysis. European Respiratory Journal. 2020;55(5):2000547. doi:10.1183/13993003.00547-2020
60. Yang J, Zheng Y, Gou X, et al. Prevalence of comorbidities and its effects in patients infected with SARS-CoV-2: a systematic review and meta-analysis. Int J Infect Dis. 2020;94:91-95. doi:10.1016/j.ijid.2020.03.017
61. Wang Y, Zhang D, Du G, Du R, Zhao J, Jin Y, et al. Remdesivir in adults with severe COVID-19: A randomised, double-blind, placebo-controlled, multicentre trial. The Lancet. 2020;395(10236):1569–78. doi:10.1016/s0140-6736(20)31022-9
62. The Novel Coronavirus Pneumonia Emergency Response Epidemiology Team. The Epidemiological Characteristics of an Outbreak of 2019 Novel Coronavirus Diseases (COVID-19) - China, 2020. China CDC Wkly. 2020;2(8):113-122.
63. Chen H, Guo J, Wang C, Luo F, Yu X, Zhang W, et al. Clinical characteristics and intrauterine vertical transmission potential of covid-19 infection in nine pregnant women: A retrospective review of medical records. The Lancet. 2020;395(10226):809–15. doi:10.1016/s0140-6736(20)30360-3
64. Grasselli G, Zangrillo A, Zanella A, Antonelli M, Cabrini L, Castelli A, et al. Baseline characteristics and outcomes of 1591 patients infected with SARS-COV-2 admitted to icus of the Lombardy region, Italy. JAMA. 2020;323(16):1574. doi:10.1001/jama.2020.5394
65. Pappa S, Ntella V, Giannakas T, Giannakoulis VG, Papoutsi E, Katsaounou P. Prevalence of depression, anxiety, and insomnia among healthcare workers during the COVID-19 pandemic: A systematic review and meta-analysis. Brain, Behavior, and Immunity. 2020;88:901–7. doi:10.1016/j.bbi.2020.05.026
66. Huang Y, Zhao N. Generalized anxiety disorder, depressive symptoms and sleep quality during COVID-19 outbreak in China: A web-based cross-sectional survey. Psychiatry Research. 2020;288:112954. doi:10.1016/j.psychres.2020.112954
67. Chinazzi M, Davis JT, Ajelli M, Gioannini C, Litvinova M, Merler S, et al. The effect of travel restrictions on the spread of the 2019 novel coronavirus (COVID-19) outbreak. Science. 2020;368(6489):395–400. doi:10.1126/science.aba9757
68. Feldstein LR, Rose EB, Horwitz SM, et al. Multisystem Inflammatory Syndrome in U.S. Children and Adolescents. N Engl J Med. 2020;383(4):334-346. doi:10.1056/NEJMoa2021680
69. To KK-W, Tsang OT-Y, Leung W-S, Tam AR, Wu T-C, Lung DC, et al. Temporal profiles of viral load in posterior oropharyngeal saliva samples and serum antibody responses during infection by SARS-COV-2: An observational cohort study. The Lancet Infectious Diseases. 2020;20(5):565–74. doi:10.1016/s1473-3099(20)30196-1
70. Jackson LA, Anderson EJ, Rouphael NG, Roberts PC, Makhene M, Coler RN, et al. An mrna vaccine against SARS-COV-2 — preliminary report. New England Journal of Medicine. 2020;383(20):1920–31. doi:10.1056/nejmoa2022483
71. Shang J, Wan Y, Luo C, Ye G, Geng Q, Auerbach A, et al. Cell entry mechanisms of SARS-COV-2. Proceedings of the National Academy of Sciences. 2020;117(21):11727–34. doi:10.1073/pnas.2003138117
72. Zhang L, Lin D, Sun X, Curth U, Drosten C, Sauerhering L, et al. Crystal structure of SARS-COV-2 main protease provides a basis for design of improved α-ketoamide inhibitors. Science. 2020;368(6489):409–12. doi:10.1126/science.abb3405
73. Ou X, Liu Y, Lei X, Li P, Mi D, Ren L, et al. Characterization of spike glycoprotein of SARS-COV-2 on virus entry and its immune cross-reactivity with SARS-COV. Nature Communications. 2020;11(1). doi:10.1038/s41467-020-15562-9
74. Li R, Pei S, Chen B, Song Y, Zhang T, Yang W, et al. Substantial undocumented infection facilitates the rapid dissemination of novel coronavirus (SARS-COV-2). Science. 2020;368(6490):489–93. doi:10.1126/science.abb3221
75. Flaxman S, Mishra S, Gandy A, Unwin HJ, Mellan TA, Coupland H, et al. Estimating the effects of non-pharmaceutical interventions on COVID-19 in Europe. Nature. 2020;584(7820):257–61. doi:10.1038/s41586-020-2405-7
76. Zhang J, Dong X, Cao Y, Yuan Y, Yang Y, Yan Y, et al. Clinical characteristics of 140 patients infected with SARS‐COV‐2 in Wuhan, China. Allergy. 2020;75(7):1730–41. doi:10.1111/all.14238
77. He X, Lau EH, Wu P, Deng X, Wang J, Hao X, et al. Temporal Dynamics in viral shedding and transmissibility of COVID-19. 2020; doi:10.1101/2020.03.15.20036707
78. Wölfel R, Corman VM, Guggemos W, Seilmaier M, Zange S, Müller MA, et al. Virological assessment of hospitalized patients with Covid-2019. Nature. 2020;581(7809):465–9. doi:10.1038/s41586-020-2196-x
79. Kampf G, Todt D, Pfaender S, Steinmann E. Persistence of coronaviruses on inanimate surfaces and their inactivation with biocidal agents. Journal of Hospital Infection. 2020;104(3):246–51. doi:10.1016/j.jhin.2020.01.022
80. Gordon DE, Jang GM, Bouhaddou M, Xu J, Obernier K, White KM, et al. A SARS-COV-2 protein interaction map reveals targets for drug repurposing. Nature. 2020;583(7816):459–68. doi:10.1038/s41586-020-2286-9
81. Hale, T., Angrist, N., Goldszmidt, R. et al. A global panel database of pandemic policies (Oxford COVID-19 Government Response Tracker). Nat Hum Behav 5, 529–538 (2021). <https://doi.org/10.1038/s41562-021-01079-8>
82. Rajkumar RP. Covid-19 and mental health: A review of the existing literature. Asian Journal of Psychiatry. 2020;52:102066. doi:10.1016/j.ajp.2020.102066
83. Lopez Bernal J, Andrews N, Gower C, Gallagher E, Simmons R, Thelwall S, et al. Effectiveness of covid-19 vaccines against the B.1.617.2 (delta) variant. New England Journal of Medicine. 2021;385(7):585–94. doi:10.1056/nejmoa2108891
84. Bikdeli B, Madhavan MV, Jimenez D, Chuich T, Dreyfus I, Driggin E, et al. Covid-19 and thrombotic or thromboembolic disease: Implications for prevention, antithrombotic therapy, and follow-up. Journal of the American College of Cardiology. 2020;75(23):2950–73. doi:10.1016/j.jacc.2020.04.031
85. Harvey, W.T., Carabelli, A.M., Jackson, B. et al. SARS-CoV-2 variants, spike mutations and immune escape. Nat Rev Microbiol 19, 409–424 (2021). <https://doi.org/10.1038/s41579-021-00573-0>
86. Jin Z, Du X, Xu Y, Deng Y, Liu M, Zhao Y, et al. Structure of mpro from SARS-COV-2 and discovery of its inhibitors. Nature. 2020;582(7811):289–93. doi:10.1038/s41586-020-2223-y
87. Docherty AB, Harrison EM, Green CA, Hardwick HE, Pius R, Norman L, et al. Features of 20133 UK patients in hospital with covid-19 using the ISARIC who clinical characterisation protocol: Prospective observational cohort study. BMJ. 2020;m1985. doi:10.1136/bmj.m1985
88. Lu X, Zhang L, Du H, Zhang J, Li YY, Qu J, et al. SARS-COV-2 infection in children. New England Journal of Medicine. 2020;382(17):1663–5. doi:10.1056/nejmc2005073
89. Fang Y, Zhang H, Xie J, Lin M, Ying L, Pang P, et al. Sensitivity of chest CT for covid-19: Comparison to RT-PCR. Radiology. 2020;296(2). doi:10.1148/radiol.2020200432
90. Wang Q, Zhang Y, Wu L, Niu S, Song C, Zhang Z, et al. Structural and functional basis of SARS-COV-2 entry by using human ACE2. Cell. 2020;181(4). doi:10.1016/j.cell.2020.03.045
91. Hadjadj J, Yatim N, Barnabei L, et al. Impaired type I interferon activity and inflammatory responses in severe COVID-19 patients. Science. 2020;369(6504):718-724. doi:10.1126/science.abc6027
92. Ye Q, Wang B, Mao J. The pathogenesis and treatment of the `cytokine storm’ in covid-19. Journal of Infection. 2020;80(6):607–13. doi:10.1016/j.jinf.2020.03.037
93. Vindegaard N, Benros ME. COVID-19 pandemic and mental health consequences: Systematic review of the current evidence. Brain Behav Immun. 2020;89:531-542. doi:10.1016/j.bbi.2020.05.048
94. Emanuel EJ, Persad G, Upshur R, Thome B, Parker M, Glickman A, et al. Fair allocation of scarce medical resources in the time of covid-19. New England Journal of Medicine. 2020;382(21):2049–55. doi:10.1056/nejmsb2005114
95. Xu X-W, Wu X-X, Jiang X-G, Xu K-J, Ying L-J, Ma C-L, et al. Clinical findings in a group of patients infected with the 2019 novel coronavirus (SARS-COV-2) outside of Wuhan, China: Retrospective case series. BMJ. 2020;m606. doi:10.1136/bmj.m606
96. Gupta A, Madhavan MV, Sehgal K, et al. Extrapulmonary manifestations of COVID-19. Nat Med. 2020;26(7):1017-1032. doi:10.1038/s41591-020-0968-3
97. Helms J, Tacquard C, Severac F, Leonard-Lorant I, Ohana M, Delabranche X, et al. High risk of thrombosis in patients with severe SARS-COV-2 infection: A multicenter prospective cohort study. Intensive Care Medicine. 2020;46(6):1089–98. doi:10.1007/s00134-020-06062-x
98. Shereen MA, Khan S, Kazmi A, Bashir N, Siddique R. Covid-19 infection: Emergence, transmission, and characteristics of human coronaviruses. Journal of Advanced Research. 2020;24:91–8. doi:10.1016/j.jare.2020.03.005
99. WHO Solidarity Trial Consortium, Pan H, Peto R, et al. Repurposed Antiviral Drugs for Covid-19 - Interim WHO Solidarity Trial Results. N Engl J Med. 2021;384(6):497-511. doi:10.1056/NEJMoa2023184
100. Wynants L, Van Calster B, Collins GS, et al. Prediction models for diagnosis and prognosis of covid-19: systematic review and critical appraisal [published correction appears in BMJ. 2020 Jun 3;369:m2204]. BMJ. 2020;369:m1328. Published 2020 Apr 7. doi:10.1136/bmj.m1328
