# Supplementary material for: The 100 most-cited articles in COVID-19: a bibliometric analysis
Source: Eur J Public Health. 2024 Jul 6;34(4):744–52. doi: 10.1093/eurpub/ckae098 (PMC11293834; doi:10.1093/eurpub/ckae098)
Supplement: ckae098_Supplementary_Data [file ckae098_supplementary_data.zip › ckae098_Supplementary_Data/ejph-2023-10-om-0567-File008.docx]

| Current rank | Previous ranking (If available) | Authors | Journals | Total number of citations | Mean citations per year | Level of evidence |
| --- | --- | --- | --- | --- | --- | --- |
| 1 | 2 | Zhou F. et al. | LANCET | 16610 | 4152.5 | 3 |
| 2 | 3 | Hoffmann M. | CELL | 11947 | 2986.75 | 5 |
| 3 | 1 | Guan W. et al. | NEW ENGLAND JOURNAL OF MEDICINE | 10146 | 2593.75 | 3 |
| 4 | 4 | Polack F.P et al. | NEW ENGLAND JOURNAL OF MEDICINE | 9013 | 2352.25 | 2 |
| 5 | 6 | The RECOVERY Collaborative Group | NEW ENGLAND JOURNAL OF MEDICINE | 6725 | 2241.67 | 2 |
| 6 | 10 | Baden L.R et al. | NEW ENGLAND JOURNAL OF MEDICINE | 6216 | 2072 | 2 |
| 7 | 9 | Walls A.C et al. | CELL | 5776 | 1444 | 5 |
| 8 | 8 | Mao L. et al. | JAMA NEUROLOGY | 5439 | 1359.75 | 4 |
| 9 | 12 | Coronaviridae Study Group of the International Committee on Taxonomy of Viruses | NATURE MICROBIOLOGY | 5042 | 1260.5 | 5 |
| 10 | 5 | Long Q.X. et al. | NATURE MEDICINE | 5036 | 1259 | 4 |
| 11 | 11 | Wu C. et al. | JAMA INTERNAL MEDICINE | 5021 | 1255.25 | 3 |
| 12 | 18 | Wang C. et al. | INTERNATIONAL JOURNAL OF ENVIRONMENTAL RESEARCH AND PUBLIC HEALTH | 4957 | 1239.25 | 4 |
| 13 | 13 | Yang X. et al. | LANCET RESPIRATORY MEDICINE | 4872 | 1218 | 3 |
| 14 | 14 | Beigel J.H et al. | NEW ENGLAND JOURNAL OF MEDICINE | 4574 | 1143.5 | 2 |
| 15 | 16 | Lai J. et al. | JAMA NETWORK OPEN | 4294 | 1073.5 | 4 |
| 16 | 7 | Bai Y. et al. | JAMA-JOURNAL OF THE AMERICAN MEDICAL ASSOCIATION | 4140 | 1035 | 5 |
| 17 | 21 | Ruan Q. et al. | INTENSIVE CARE MEDICINE | 3968 | 992 | 3 |
| 18 | 25 | Lan J. et al. | NATURE | 3812 | 953 | 5 |
| 19 | 51 | Dhama K. et al. | CLINICAL MICROBIOLOGY REVIEWS | 3776 | 944 | 5 |
| 20 | 15 | Richardson S. et al. | JAMA-JOURNAL OF THE AMERICAN MEDICAL ASSOCIATION | 3727 | 931.75 | 4 |
| 21 | 28 | Williamson E.J et al. | NATURE | 3659 | 914.75 | 3 |
| 22 | 27 | Ackermann M et al. | NEW ENGLAND JOURNAL OF MEDICINE | 3605 | 901.25 | 5 |
| 23 | 24 | Lauer S.A et al. | ANNALS OF INTERNAL MEDICINE | 3444 | 861 | 3 |
| 24 | 31 | Holmes E.A et al. | LANCET PSYCHIATRY | 3350 | 837.5 | 5 |
| 25 | 22 | Cao B. et al. | NEW ENGLAND JOURNAL OF MEDICINE | 3335 | 833.75 | 2 |
| 26 | 33 | Nicola M. et al. | INTERNATIONAL JOURNAL OF SURGERY | 3332 | 833 | 5 |
| 27 | 29 | Qin C. et al. | CLINICAL INFECTIOUS DISEASES | 3295 | 823.75 | 3 |
| 28 | 30 | Yan R. et al. | SCIENCE | 3269 | 817.25 | 5 |
| 29 | 26 | Gautret P. et al. | INTERNATIONAL JOURNAL OF ANTIMICROBIAL AGENTS | 3117 | 779.25 | 3 |
| 30 | 34 | Chen G. et al. | JOURNAL OF CLINICAL INVESTIGATION | 3061 | 764.25 | 3 |
| 31 | 42 | Wiersinga W.J et al. | JAMA-JOURNAL OF THE AMERICAN MEDICAL ASSOCIATION | 3027 | 756.75 | 5 |
| 32 | 36 | Sohrabi C. et al. | INTERNATIONAL JOURNAL OF SURGERY | 3018 | 754.5 | 5 |
| 33 | 32 | Liang W. et al. | LANCET ONCOLOGY | 2961 | 740.25 | 3 |
| 34 | 47 | Voiysey M et al. | LANCET | 2897 | 965.67 | 2 |
| 35 | 46 | Cao W. et al. | PSYCHIATRY RESEARCH | 2860 | 715 | 5 |
| 36 | 54 | Xiong J et al. | JOURNAL OF AFFECTIVE DISORDERS | 2849 | 712.25 | 1 |
| 37 | 39 | Rothan H.A., Byrareddy S.N. | JOURNAL OF AUTOIMMUNITY | 2843 | 710.75 | 5 |
| 38 | 23 | Xu Z. et al. | LANCET RESPIRATORY MEDICINE | 2823 | 705.75 | 5 |
| 39 | 44 | Tay M.Z et al. | NATURE REVIEWS IMMUNOLOGY | 2780 | 695 | 5 |
| 40 | 50 | Bavel J.J et al. | NATURE HUMAN BEHAVIOUR | 2759 | 689.75 | 5 |
| 41 | 75 | Hu B. et al. | NATURE REVIEWS MICROBIOLOGY | 2741 | 913.67 | 5 |
| 42 | 41 | Shi S. et al. | JAMA CARDIOLOGY | 2705 | 676.25 | 3 |
| 43 | 49 | Blanco-Melo D. et al. | CELL | 2677 | 669.25 | 5 |
| 44 | 40 | WHO | PEDIATRIA I MEDYCYNA RODZINNA-PAEDIATRICS AND FAMILY MEDICINE | 2664 | 666 | 5 |
| 45 | 45 | Guo T. et al. | JAMA CARDIOLOGY | 2618 | 654.5 | 4 |
| 46 | 17 | Klok F.A et al. | THROMBOSIS RESEARCH | 2590 | 647.5 | 3 |
| 47 | 35 | Lai C.C et al. | INTERNATIONAL JOURNAL OF ANTIMICROBIAL AGENTS | 2579 | 644.75 | 5 |
| 48 | 43 | Chen T et al. | BMJ-BRITISH MEDICAL JOURNAL | 2577 | 644.25 | 4 |
| 49 | 19 | Tao Ai et al. | RADIOLOGY | 2566 | 641.5 | 3 |
| 50 | 62 | Ahorsu D. K et al. | INTERNATIONAL JOURNAL OF MENTAL HEALTH AND ADDICTION | 2515 | 1257.5 | 3 |
| 51 | 52 | Korber B et al. | CELL | 2493 | 623.25 | 5 |
| 52 | 48 | Guo Y.R et al. | MILITARY MEDICAL RESEARCH | 2493 | 623.25 | 5 |
| 53 | 38 | Dong Y et al. | PEDIATRICS | 2475 | 618.75 | 3 |
| 54 | 55 | Grifoni A. et al. | CELL | 2420 | 605 | 5 |
| 55 | 57 | Chu D.K et al. | LANCET | 2407 | 601.75 | 1 |
| 56 | 56 | Shang J et al. | NATURE | 2357 | 589.25 | 5 |
| 57 | 95 | Khoury D.S et al. | NATURE MEDICINE | 2350 | 783.33 | 5 |
| 58 | NEW | Nalbandian A. et al. | NATURE MEDICINE | 2337 | 779 | 5 |
| 59 | 37 | Guan W. et al. | EUROPEAN RESPIRATORY JOURNAL | 2277 | 569.25 | 3 |
| 60 | NEW | Wang Y.M. et al. | INTERNATIONAL JOURNAL OF INFECTIOUS DISEASES | 2266 | 566.5 | 1 |
| 61 | 60 | Wang Y. et al. | LANCET | 2208 | 552 | 2 |
| 62 | NEW | Feng Z.J. et al. | CHINA CDC WEEKLY | 2203 | 550.75 | 5 |
| 63 | 53 | Chen H. et al. | LANCET | 2193 | 548.25 | 4 |
| 64 | 73 | Grasselli G. et al. | JAMA-JOURNAL OF THE AMERICAN MEDICAL ASSOCIATION | 2140 | 535 | 4 |
| 65 | 83 | Pappa S. et al. | BRAIN BEHAVIOR AND IMMUNITY | 2138 | 534.5 | 1 |
| 66 | 72 | Huang Y. and Zhao N. | PSYCHIATRY RESEARCH | 2127 | 531.75 | 4 |
| 67 | 74 | Chinazzi M. et al. | SCIENCE | 2112 | 528 | 5 |
| 68 | NEW | Feldstein L.R. et al. | NEW ENGLAND JOURNAL OF MEDICINE | 2108 | 527 | 3 |
| 69 | 61 | To K.K.W et al. | LANCET INFECTIOUS DISEASES | 2096 | 524 | 3 |
| 70 | 78 | Jackson L. A et al. | NEW ENGLAND JOURNAL OF MEDICINE | 2087 | 521.75 | 5 |
| 71 | 81 | Shang J. et al. | PROCEEDINGS OF THE NATIONAL ACADEMY OF SCIENCES OF THE UNITED STATES OF AMERICA | 2082 | 520.5 | 5 |
| 72 | 80 | Zhang L. et al. | SCIENCE | 2067 | 516.75 | 5 |
| 73 | 66 | Ou X. et al. | NATURE COMMUNICATIONS | 2053 | 513.25 | 5 |
| 74 | 64 | Li R. et al. | SCIENCE | 2036 | 509 | 5 |
| 75 | 96 | Flaxman S. et al. | NATURE | 2026 | 506.5 | 5 |
| 76 | 88 | Zhang J. et al. | ALLERGY | 2011 | 502.75 | 3 |
| 77 | 71 | He X. et al. | NATURE MEDICINE | 2007 | 501.75 | 5 |
| 78 | 70 | Wolfel R. et al. | NATURE | 2003 | 500.75 | 4 |
| 79 | 69 | Kampf G. et al. | JOURNAL OF HOSPITAL INFECTION | 1999 | 499.75 | 5 |
| 80 | 85 | Gordon D. E et al. | NATURE | 1998 | 499.5 | 5 |
| 81 | NEW | Hale T. et al. | NATURE HUMAN BEHAVIOUR | 1989 | 663 | 5 |
| 82 | 79 | Rajkumar R. P. | ASIAN JOURNAL OF PSYCHIATRY | 1988 | 497 | 5 |
| 83 | 97 | Lopez Bemal J. | NEW ENGLAND JOURNAL OF MEDICINE | 1967 | 655.67 | 4 |
| 84 | 77 | Bikdeli B. et al. | JOURNAL OF THE AMERICAN COLLEGE OF CARDIOLOGY | 1959 | 489.75 | 5 |
| 85 | NEW | Harvey W.T. et al. | NATURE REVIEWS MICROBIOLOGY | 1957 | 652.33 | 5 |
| 86 | 59 | Jin Z. et al. | NATURE | 1950 | 487.5 | 5 |
| 87 | 89 | Docherty A.B et al. | BMJ-BRITISH MEDICAL JOURNAL | 1947 | 486.75 | 3 |
| 88 | 65 | Lu X. et al. | NEW ENGLAND JOURNAL OF MEDICINE | 1914 | 478.5 | 3 |
| 89 | 76 | Fang Y. et al. | RADIOLOGY | 1908 | 477 | 3 |
| 90 | 82 | Wang Q. et al. | CELL | 1888 | 472 | 5 |
| 91 | NEW | Hadjadj J. et al. | SCIENCE | 1877 | 469.25 | 5 |
| 92 | 91 | Ye Q. et al. | JOURNAL OF INFECTION | 1869 | 467.25 | 5 |
| 93 | NEW | Vindegaard N. et al. | BRAIN BEHAVIOR AND IMMUNITY | 1859 | 464.75 | 1 |
| 94 | 90 | Emanuel E. J et al. | NEW ENGLAND JOURNAL OF MEDICINE | 1835 | 458.75 | 5 |
| 95 | 67 | Xu X. W et al. | BMJ-BRITISH MEDICAL JOURNAL | 1810 | 452.5 | 4 |
| 96 | NEW | Gupta A. et al. | NATURE MEDICINE | 1805 | 451.25 | 5 |
| 97 | 93 | Helms J. et al. | INTENSIVE CARE MEDICINE | 1797 | 449.25 | 3 |
| 98 | 99 | Shereen M.A et al. | JOURNAL OF ADVANCED RESEARCH | 1788 | 447 | 5 |
| 99 | NEW | WHO Solidarity Trial Consortium | NEW ENGLAND JOURNAL OF MEDICINE | 1769 | 589.67 | 2 |
| 100 | NEW | Wynants L et al. | BMJ-BRITISH MEDICAL JOURNAL | 1765 | 441.25 | 1 |

**Appendix 3: Table showing the 100 most-cited articles searched in 2024**
